# Mothers after Gestational Diabetes in Australia (MAGDA): A Randomised Controlled Trial of a Postnatal Diabetes Prevention Program

**DOI:** 10.1371/journal.pmed.1002092

**Published:** 2016-07-26

**Authors:** Sharleen L. O’Reilly, James A. Dunbar, Vincent Versace, Edward Janus, James D. Best, Rob Carter, Jeremy J. N. Oats, Timothy Skinner, Michael Ackland, Paddy A. Phillips, Peter R. Ebeling, John Reynolds, Sophy T. F. Shih, Virginia Hagger, Michael Coates, Carol Wildey, MAGDA Study Group

**Affiliations:** 1 Institute of Physical Activity and Nutrition, Deakin University, Burwood, Victoria, Australia; 2 Centre for Population Health Research, Faculty of Health, Deakin University, Burwood, Victoria, Australia; 3 School of Medicine, Deakin University, Warrnambool, Victoria, Australia; 4 Department of Medicine, Melbourne Medical School–Western Precinct, University of Melbourne, St Albans, Victoria, Australia; 5 General Internal Medicine Unit, Western Health, Sunshine Hospital, St Albans, Victoria, Australia; 6 Lee Kong Chian School of Medicine, Imperial College London and Nanyang Technological University, Singapore; 7 Melbourne School of Population and Global Health, University of Melbourne, Parkville, Victoria, Australia; 8 School of Psychological and Clinical Sciences, Charles Darwin University, Casuarina, Northern Territory, Australia; 9 Department of Epidemiology and Preventive Medicine, Monash University, Clayton, Victoria, Australia; 10 Department of Medicine, Flinders University, Bedford Park, South Australia, Australia; 11 SA Health, Adelaide, South Australia, Australia; 12 Department of Medicine, School of Clinical Sciences, Monash University, Clayton, Victoria, Australia; 13 Alfred Health and Faculty of Medicine, Nursing and Health Sciences, Monash University, Melbourne, Victoria, Australia; 14 Diabetes Australia Victoria, Melbourne, Victoria, Australia; University of Cambridge, UNITED KINGDOM

## Abstract

**Background:**

Gestational diabetes mellitus (GDM) is an increasingly prevalent risk factor for type 2 diabetes. We evaluated the effectiveness of a group-based lifestyle modification program in mothers with prior GDM within their first postnatal year.

**Methods and Findings:**

In this study, 573 women were randomised to either the intervention (*n* = 284) or usual care (*n* = 289). At baseline, 10% had impaired glucose tolerance and 2% impaired fasting glucose. The diabetes prevention intervention comprised one individual session, five group sessions, and two telephone sessions. Primary outcomes were changes in diabetes risk factors (weight, waist circumference, and fasting blood glucose), and secondary outcomes included achievement of lifestyle modification goals and changes in depression score and cardiovascular disease risk factors. The mean changes (intention-to-treat [ITT] analysis) over 12 mo were as follows: −0.23 kg body weight in intervention group (95% CI −0.89, 0.43) compared with +0.72 kg in usual care group (95% CI 0.09, 1.35) (change difference −0.95 kg, 95% CI −1.87, −0.04; group by treatment interaction *p* = 0.04); −2.24 cm waist measurement in intervention group (95% CI −3.01, −1.42) compared with −1.74 cm in usual care group (95% CI −2.52, −0.96) (change difference −0.50 cm, 95% CI −1.63, 0.63; group by treatment interaction *p* = 0.389); and +0.18 mmol/l fasting blood glucose in intervention group (95% CI 0.11, 0.24) compared with +0.22 mmol/l in usual care group (95% CI 0.16, 0.29) (change difference −0.05 mmol/l, 95% CI −0.14, 0.05; group by treatment interaction *p* = 0.331). Only 10% of women attended all sessions, 53% attended one individual and at least one group session, and 34% attended no sessions. Loss to follow-up was 27% and 21% for the intervention and control groups, respectively, primarily due to subsequent pregnancies. Study limitations include low exposure to the full intervention and glucose metabolism profiles being near normal at baseline.

**Conclusions:**

Although a 1-kg weight difference has the potential to be significant for reducing diabetes risk, the level of engagement during the first postnatal year was low. Further research is needed to improve engagement, including participant involvement in study design; it is potentially more effective to implement annual diabetes screening until women develop prediabetes before offering an intervention.

**Trial Registration:**

Australian New Zealand Clinical Trials Registry ACTRN12610000338066

## Abstract 2

Sharleen O'Reilly and colleagues investigate the effectiveness of a diabetes prevention program for reducing weight, waist circumference and fasting glucose measurements for women who have had gestational diabetes.

## Author Summary

**Why Was This Study Done?:**

- Women who have had gestational diabetes are much more likely to develop type 2 diabetes.
- Although many diabetes prevention programs for people over the age of 50 exist, few are tailored to the needs of young mothers who have had gestational diabetes.
- On the assumption that offering prevention earlier is beneficial, researchers developed and tested a diabetes prevention program for women who had gestational diabetes; women participated in the program during their first year after giving birth.

**What Did the Researchers Do and Find?:**

- The researchers enrolled 573 women in a one-year study: 284 women were assigned to the diabetes prevention program (one individual session and five group sessions over a three-month period, followed by telephone calls at six and nine months), and 289 were assigned to the control group (usual postnatal care).
- After one year, the average changes for women in the diabetes prevention program were a 0.23-kg decrease in weight, a 2.24-cm decrease in waist circumference, and a 0.18-mmol/l increase in fasting blood glucose, while the average changes for women in the control group were a 0.72-kg increase in weight, a 1.74-cm decrease in waist circumference, and a 0.22-mmol/l increase in fasting blood glucose. The between-group difference in weight change was 0.95 kg.
- The number of women who attended the diabetes prevention program was lower than anticipated—10% attended all sessions, and 53% attended the individual session plus at least one group session; about a quarter of women did not complete the study, mainly due to becoming pregnant again.

**What Do These Findings Mean?:**

- These findings suggest that although a diabetes prevention program designed for women who have had gestational diabetes can prevent weight gain over 12 months, getting women to engage with the program was challenging, so it would not be sustainable in routine health services.
- The women who participated in the study had low diabetes risk profiles (only one in ten had impaired glucose tolerance), and most diabetes prevention guidelines would not categorise them as being at sufficiently high risk for participation in a diabetes prevention program.
- For diabetes prevention programs in women who have had gestational diabetes, further research is required on the process of engagement and lifestyle interventions at other time points, including participant involvement in the design of interventions. Australian clinical guidelines stipulate that women who have had gestational diabetes should be screened annually for diabetes. One option for management would be to wait until they develop prediabetes before offering a diabetes prevention program, which may prove more effective because their children will be older and women may be easier to engage in improving their health.

## Introduction

Gestational diabetes mellitus (GDM) and type 2 diabetes mellitus (T2DM) rates are rising worldwide [1], posing an increasing burden on the health and economic welfare of nations [2]. Women with GDM are seven times more likely to develop T2DM than women who have normoglycemic pregnancies [3]. Diabetes prevention is possible; two landmark studies in high risk individuals from the general population showed that T2DM incidence could be reduced by 58% with a combination of weight loss and moderate physical activity [4,5]. The lifestyle modification program for the original US Diabetes Prevention Program (US-DPP) was implemented over a 24-wk intensive intervention period with 16 individual coaching sessions, and a maintenance period with individual sessions every 2 mo for 24 mo [6]. Positive prevention outcomes (50% reduction in risk) were found for women with previous GDM within a subgroup analysis of the US-DPP [7].

Given that women with prior GDM are at high risk of developing T2DM and cardiovascular disease earlier in their lifespan than women with normoglycemic pregnancies [8], intervening early with a suitable diabetes prevention program (DPP) has the potential to yield positive health outcomes. Interpregnancy weight gain contributes to an increased risk of adverse pregnancy outcomes for both mother and baby during subsequent pregnancies [9,10], and the children of women with GDM are at increased risk of obesity and T2DM later in life [8]. Reducing postpartum weight retention decreases perinatal complications [10] and T2DM risk [11,12] and can influence the health status of a woman’s children [13,14], but designing interventions for this life stage is challenging [15,16]. It is well recognised that many barriers exist to mothers engaging in behaviour change during the early infancy period, including tiredness, lack of time, competing work and carer duties, and cultural expectations [17–19]. It is unclear the extent to which these obstacles can be overcome with a lifestyle modification program specifically designed to meet the needs of this population, as trials thus far have reported inconsistent results [7,20–26].

The Mothers After Gestational Diabetes in Australia Diabetes Prevention Program (MAGDA-DPP) study was undertaken to test the effectiveness of a group-based lifestyle modification program offered in the first postnatal year to women with previous GDM. At the time of MAGDA-DPP design, evidence on the optimal intervention was gathered [27], and Greater Green Triangle Diabetes Prevention Program (GGT-DPP) [28] content was adapted to meet known barriers and characteristics of the participant life stage. The program aimed to promote changes, relative to usual care, in anthropometric, behavioural, and biomedical outcomes [29,30].

## Methods

### Study Design

MAGDA-DPP was a multicentre, prospective, open randomised controlled trial (RCT) to assess the effectiveness of a structured DPP for women with previous GDM. The detailed methods and research design of MAGDA-DPP are described elsewhere [29,30]. The trial recruited women from two Australian state capital cities, Melbourne and Adelaide. The study was approved by the relevant ethics committees and registered prospectively as an RCT. The co-primary outcomes for MAGDA-DPP were change in fasting blood glucose, waist circumference, and weight at 12 mo.

### Participants

Women aged ≥18 y with a diagnosis of GDM in their most recent pregnancy were eligible for inclusion. GDM was defined by Australasian Diabetes in Pregnancy Society (ADIPS) criteria [31] at the time of study commencement: fasting plasma glucose (FPG) of 5.5 mmol/l or higher, or 2-h glucose of 8.0 mmol/l or higher on a 75-g oral glucose tolerance test (OGTT), or a glucose challenge test result of 11.1 mmol/l or higher. Exclusion criteria were the following: preexisting diabetes (type 1 diabetes mellitus or T2DM); cancer (not in remission); severe mental illness; substance abuse (illicit drugs); myocardial infarction in the preceding 3 mo; difficulty with English; involvement in another postnatal intervention trial; and pregnancy at postnatal baseline testing or at any point during the 12 mo of study involvement. Women diagnosed with T2DM or who became pregnant during the study were excluded based on the influence their condition would have on the primary outcome measures of weight, waist circumference, and FPG.

### Recruitment

MADGA-DPP used multiple recruitment strategies, prospective and retrospective, which are described in full within our methodology publications [29,30]. Briefly, prospective recruitment involved approaching women in the antenatal clinic soon after the diagnosis of GDM, at around 28 wk of pregnancy, and conducting eligibility screening. Eligible women were provided with a patient information and consent form to return via prepaid envelope within 4 wk. If consent forms were not received within that time frame, follow-up contact was made with the woman. Once consent was received, the woman was contacted at 3 mo postpartum to initiate baseline testing.

The National Diabetes Services Scheme (NDSS) is an Australian Government initiative to subsidise blood glucose monitoring supplies for people with diabetes and is in effect a national diabetes registry. Women who have had GDM are recorded in a subregistry of the NDSS called the National Gestational Diabetes Register (NGDR). Retrospective recruitment occurred using the following approaches: a mail-out via the NDSS (using data from the NGDR) to women living in relevant postcodes in Adelaide (South Australia) and Melbourne (Victoria); referrals from one private obstetrician (South Australia); and hospital records database mining (South Australia). Women who had GDM diagnosed during their most recent pregnancy were approached and screened for eligibility using the predefined inclusion and exclusion criteria. Written informed consent was obtained from all participants, regardless of recruitment method, once screening confirmed their eligibility. The MAGDA-DPP commenced recruitment 17 January 2011, and the last participant completed final testing 28 May 2015.

### Data Collection

Following signed consent, a trained research nurse conducted the study assessments in the participant’s home. All participants completed a baseline assessment, and the assessment was repeated after 12 mo. Standardised protocols for all clinical measures (blood pressure, anthropometry, blood sampling) were implemented [32]. Blood samples were collected by the study nurse/phlebotomist and analysed by Melbourne Pathology (Victoria) or Clinpath Laboratories (South Australia). The study nurse conducted the anthropometric (height, weight, waist circumference) and blood pressure measurements. Women provided fasting venous samples for lipid (triglycerides, total cholesterol, low-density lipoprotein cholesterol [LDL-C] and high-density lipoprotein cholesterol [HDL-C]), HbA1c, and glucose (fasting and 2-h OGTT) analysis. In addition to the baseline and 12-mo assessments, the intervention group repeated all blood tests (except OGTT) and weight and waist measures at 3 mo after baseline testing or as soon as possible after their final MAGDA-DPP group session. Survey data were completed at baseline and 12 mo and included the questions about the following: demographics (breastfeeding, parity, education, employment status, cultural background; baseline only); diet (Food Frequency Questionnaire [33]); physical activity (Active Australia Questionnaire [34]); self-regulation and self-efficacy for diet and physical activity (not reported); social support (Multidimensional Scale of Perceived Social Support; not reported); quality of life (Assessment of Quality of Life 8D; not reported); depressive symptoms and suicidal ideation (Patient Health Questionnaire 9 [PHQ-9]); and health status (including smoking status and history of diabetes, myocardial infarction, cancer, and mental disorders). Health status information on history of diabetes, myocardial infarction, cancer, and mental disorders was collected at baseline for checking of exclusion criteria. All women completing clinical testing were provided with a standardised feedback letter on their test results, and a copy was sent to their nominated family physician.

The MAGDA-DPP adopted the lifestyle modification goals of the Finnish Diabetes Prevention Study (FIN-DPS) [35] for the intervention program content and messaging. The MAGDA-DPP had five lifestyle modification goals: ≤30% of energy from fat, ≤10% of energy from saturated fat, ≥15 g dietary fibre per 1,000 kcal, ≥30 min of moderate physical activity daily, and ≥5% body weight reduction. The first three goals were calculated using Food Frequency Questionnaire data [33], the fourth using Active Australia Questionnaire data [34], and the fifth using actual body weight. In the FIN-DPS, the number of lifestyle modification goals achieved was inversely associated with diabetes incidence over a 13-y period [5].

### Randomisation

The trial was registered with the Australian New Zealand Clinical Trials Registry on 28 April 2010, and the first participant was randomised on 1 August 2011. Once baseline diabetes screening (OGTT) results were known, eligible women were randomly allocated into either the intervention or control arm using the MAGDA-DPP management database. Permuted block randomisation was stratified by recruitment location and method. A sequence number was displayed, and the assignment code (usual care or intervention) revealed to the user in the randomisation office at Deakin University.

### Diabetes Prevention Program

After randomisation, the active intervention consisted of one individual and five group sessions delivered by specially trained healthcare professionals, with two additional follow-up maintenance telephone calls for each participant, as shown in S1 Fig. As previously described [29,30], the lifestyle intervention was informed by a theoretical framework based on the Health Action Process Approach and supported by social cognitive and self-regulation theory [36]. The intervention was based on the GGT-DPP, which was previously shown to be effective in producing change in diabetes risk factors [28]. The MAGDA-DPP was tailored to reflect relevant barriers for mothers of young children (for example, topics covered the impact of sleep deprivation, stress management and mindful eating, healthy eating for families, weaning, culturally appropriate and cost- and time-saving food preparation, and exercise considerations when caring for young children), and mothers were able to bring their children along to group sessions.

Fidelity measures were incorporated throughout the intervention (facilitator manual, detailed training program, and audio recording of all facilitator sessions). The first DPP session (the individual session) was delivered in the woman’s home by the facilitator, and the MAGDA-DPP handbook was provided. The session focus was on the intention formation component of the Health Action Process Approach, personalisation of T2DM risk using a risk algorithm, and individual goal setting. This was followed by five group sessions held at 2-wk intervals and two subsequent individual phone calls at 3 and 6 mo after the final group session. Each group session was approximately 2 h in duration, with up to 15 women per group. Session content details are reported in the protocol paper [30]. Women were encouraged to set and review at least one personal goal relating to diet and one relating to physical activity at each program session (Box 1). Women in the control group received usual care and were offered the intervention program after their 12-mo final assessment.

**Box 1. MAGDA-DPP Intervention Components**

**Intensive Phase**

**Individual session (delivered in participant’s home)**

DPP overview, personalised participant T2DM risk assessment, five lifestyle modification goals described, building participant commitment, personalised diet and physical activity goal setting with participant.

**Group session 1 (community venue within 1 mo of individual session)**

Understanding diabetes and diabetes risk factors, knowledge and skill building on the topic of saturated fat, family-focused activities on reducing saturated fat content in diet, review of personalised goals and group goal setting for next 2 wk.

**Group session 2 (community venue 2 wk after group session 1)**

Knowledge and skill building on modifying the total fat content of participants’ diet, discussion on postpartum weight management, learning activities focused on reducing fat content in diet for whole family, review of personalised goals and group goal setting for next 2 wk.

**Group session 3 (community venue 2 wk after group session 2)**

Knowledge and skill building on increasing the fibre content of participants’ diet, learning activities focused on healthier food shopping and getting more fibre into the whole family’s diet and meals, review of personalised goals and group goal setting for next 2 wk.

**Group session 4 (community venue 2 wk after group session 3)**

Knowledge and skill building on healthier meal planning, learning activities focused on negotiating stressful situations around food choice with family members and mindful eating, knowledge and skill building on good sleep hygiene, review of personalised goals and group goal setting for next 2 wk.

**Group session 5 (community venue 2 wk after group session 4)**

Knowledge and skill building on postnatal depression awareness and stress management, discussion on lifestyle modification relapse prevention and change maintenance, review of personalised goals and group longer-term goal setting.

**Maintenance Phase**

**Telephone session 1 (3 mo after group session 5)**

Review of progress and longer-term goal setting.

**Telephone session 2 (6 mo after group session 5)**

Review of progress and longer-term goal setting.

### Program Evaluation

The penetration, implementation, participation, and effectiveness (PIPE) framework for evaluating real-world program and product design elements important to implementation is a metric to evaluate the net impact of health improvement programs [37]. Four elements make up the PIPE metrics: penetration of the program into the population of interest; implementation of the proposed set of services; participation in the program; and effectiveness in generating expected outcomes. Penetration is defined as the number of individuals reached/invited divided by the number of individuals in the target population. According to Aziz et al. [37], penetration of 33% or lower is considered low, 34%–66% as moderate, 67% or higher as high. Program implementation is rated on three aspects: frequency of contact, duration of the intervention, and fidelity measures. Frequency of contact is defined based on the number, length, and type of contact within the first 12 mo of a program. A group or individual contact counts as one session, an online/telephone contact counts as 0.5 of a session, and a text/email/fax contact counts as 0.25 of a session. The total number of sessions is divided by the number of sessions delivered within the US-DPP (22 sessions) to calculate frequency (≤33% low, 34%–66% moderate, ≥67% high). Interventions lasting less than 6 mo are defined as low duration, 6–12 mo as moderate duration, and more than 12 mo as high duration. Fidelity is rated as follows: no standard curriculum as low, standard curriculum but no quality assurance measures reported as moderate, and a standard curriculum and quality assurance measures reported as high. Participation is the number of individuals enrolled in the intervention divided by the number of individuals reached/invited (≤33% low, 34%–66% moderate, ≥67% high). For DPPs, effectiveness is rated on three criteria: outcome success (number of participants achieving the main outcome divided by total number of participants completing intervention, where ≤25% low, 26%–40% moderate, >40% high success), average weight loss (≤2.3 kg low, 2.4–4.6 kg moderate, >4.6 kg high), and absolute/relative risk reduction (≤15% low, 16%–30% moderate, >30% high).

### Statistical Analysis

Analyses of primary and secondary endpoints were performed using SPSS version 22 and independently verified in GenStat release 16.1. Participants’ baseline characteristics are presented as summary measures. A statistical analysis plan was prepared, finalised, and signed off by the project guarantor prior to statistician unblinding. Analyses were carried out for all participants randomised to the study (ITT set, *n* = 573) and for the per protocol set (PPS, *n* = 331). The PPS analysis was confined to the subset of ITT participants excluding those with major protocol deviations such as allocation to the intervention but no exposure to any intervention sessions or ineligibility. Protocol deviations were determined independently of, and prior to, the unblinding of the trial statistician. PPS exclusions included post-baseline assessments beyond the specified time window (*n* = 2), pregnancy (*n* = 75), randomised to control group but received the intervention (*n* = 1), participation in another postnatal intervention during the trial (*n* = 3), should not have been randomised to trial (T2DM at baseline, *n* = 1), diagnosed with T2DM during trial (*n* = 11), lost contact or moved away or overseas (*n* = 48), and withdrew (*n* = 19). Similar proportions of women in the usual care and intervention arms—14% (40/289) and 12% (35/284), respectively—became pregnant during the trial. Also excluded from the PPS were women who did not receive minimum exposure to the intervention (*n* = 78). Minimum exposure was defined in the statistical analysis plan as attending the individual session and at least one group session.

Mixed model analyses of continuous scale endpoints used the residual maximum likelihood (REML) method to cope with missing values. The significance of the *F*-test for the group by time interaction is reported, as well as *t*-tests for within-group changes over time and between-group differences at each time point. The proportion of participants in each group known to have achieved each of the first four lifestyle modification goals at baseline and 12 mo was calculated, and the method of generalised estimating equations was used to fit models to enable group by time interactions to be tested (Wald chi-squared test). Lifestyle modification goal 5 (≥5% body weight reduction at 12 mo) was assessed using a two-sample binomial test to compare the proportions in each group. The number of goals achieved (0 to 5) by individuals at 12 mo was assessed using a mixed model analysis. Sensitivity analyses, in which missing assessments were deemed to indicate unmet goals, were conducted for each goal and also for the combined score. Unless otherwise stated, all statistical tests were conducted at the 5% significance level, with no adjustments for multiplicity of either endpoints or comparisons.

The required sample size, using a two-sided 5% significance level and 80% power, was 574 (287 in each arm); this was based on the co-primary endpoint with the smallest conjectured effect size, namely, the change in FPG over 12 mo in the GGT-DPP study [28], and thus the study was powered to detect an effect size of ≥0.27 mmol/l (assuming a mean difference between the intervention and control groups of 0.14 mmol/l and a within-group standard deviation of 0.5 mmol/l). The sample size was increased to allow for an attrition rate of up to 25%.

## Results

### Participants

**Fig 1:**
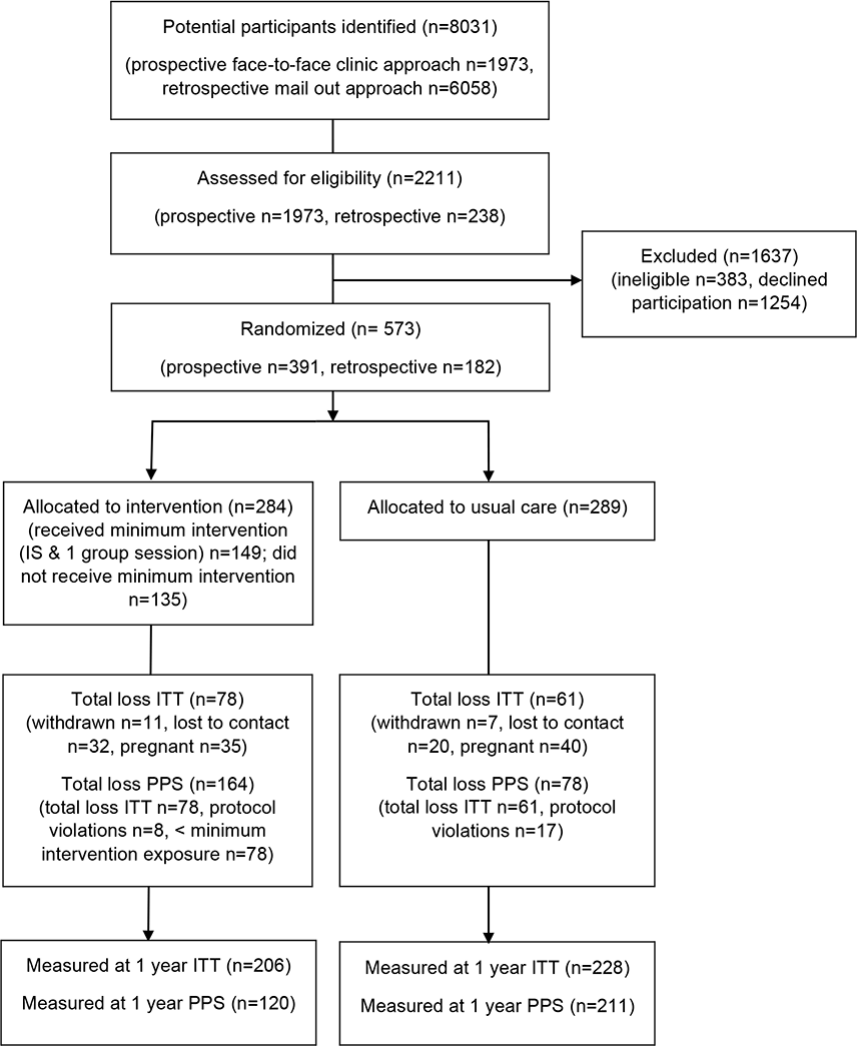
Trial flow using CONSORT reporting. ITT and PPS participants reported following randomisation. IS, individual session; loss, loss to follow-up.

We approached 8,031 women, either face-to-face or via mailed out invitation, and of those, 2,211 (28%) were screened for eligibility. Of these, 828 women (38%) consented to participate in the trial, and 573 (69%) were randomised. It took 41 mo to recruit and randomise the 573 participants. The trial flow for MAGDA-DPP is shown in Fig 1. While 28% and 38% of participants were overweight or obese, respectively, the level of impaired glucose metabolism was low in the cohort (*n* = 58 [10%] with impaired fasting glucose; *n* = 10 [2%] with impaired glucose tolerance [IGT]). The intervention and usual care groups were comparable in their baseline characteristics (Table 1). The mean age of participants’ infants at baseline was 8.0 mo (standard deviation 4.8). The number of women excluded from the PPS analysis was different between the groups (*n* = 164 [58%] intervention participants excluded; *n* = 78 [27%] control participants excluded), and this difference remained significant when exclusion for not meeting minimum program exposure was removed (*n* = 139 [49%] intervention participants excluded; *n* = 78 [27%] control participants excluded). Retention rates for the intervention and usual care groups were 73% and 79%, respectively. When pregnancy was removed from loss to follow-up data, the retention rates were 93% for the usual care group and 85% for the intervention group. A single adverse event was recorded within the study (needle stick trauma), but this occurred during screening and prior to randomisation.

**Table 1:** Baseline characteristics by treatment condition in the MAGDA-DPP study.

Characteristic	Control (*n* = 289)	Intervention (*n* = 284)	All Participants (*n* = 573)
**Age (years)**			
*N*	287	281	568
Mean (standard deviation)	33.6 (5.1)	34.1 (5.3)	33.8 (5.2)
**Waist circumference (cm)**			
*N*	289	283	572
Mean (standard deviation)	90.4 (14.5)	92.1 (14.4)	91.2 (14.5)
**BMI**			
*N*	288	281	569
Mean (standard deviation)	28.4 (6.7)	29.2 (6.9)	28.8 (6.8)
**Weight (kg)**			
*N*	289	284	573
Mean (standard deviation)	74.6 (20.3)	76.7 (20.0)	75.6 (20.2)
**Physical activity (min/d)**	35.1 (42.9)	33.4 (43.7)	34.3 (42.9)
**Energy intake (kcal/d)**	1,899 (860)	1,860 (799)	1,880 (816)
**Energy from total fat (percent/d)**	37.9 (5.2)	38.1 (5.3)	38.0 (5.2)
**Fibre intake (g/d)**	20.6 (8.8)	20.4 (8.2)	20.5 (8.5)
**Haemoglobin A1c (percent)**			
*N*	289	283	572
Mean (standard deviation)	5.34 (0.45)	5.34 (0.39)	5.34 (0.42)
**OGTT (mmol/l)**			
*N*	289	282	571
Fasting glucose	4.72 (0.52)	4.82 (0.54)	4.77 (0.53)
2-h glucose	5.55 (1.60)	5.53 (1.67)	5.54 (1.63)
**Tertiary education**	237 (82.6%)	237 (84.3%)	474 (83.5%)
**Income**			
Low	57 (20.0%)	71 (25.5%)	128 (22.7%)
Medium	133 (46.7%)	119 (42.8%)	252 (44.8%)
High	95 (33.3%)	88 (31.7%)	183 (32.5%)
**Current smoker**	21 (7.3%)	11 (3.9%)	32 (5.6%)
**Full-time employed**	49 (17.0%)	46 (16.2%)	95 (16.6%)
**Depressive symptoms (PHQ-9)**			
Minimal depression (score 0–9)	254 (88.8%)	256 (90.5%)	510 (89.6%)
Moderate depression (score 10–19)	31 (10.8%)	27 (9.5%)	58 (10.2%)
Severe depression (score 20–27)	1 (0.3%)	0 (0.0%)	1 (0.2%)
**Breastfeeding initiated**	259 (90.2%)	238 (84.7%)	497 (87.5%)
**Parity**			
1	126 (43.9%)	127 (45.2%)	253 (44.5%)
2	97 (33.8%)	97 (34.5%)	194 (34.2%)
3+	64 (22.3%)	57 (20.3%)	121 (21.3%)
**Cultural background by geo-region**			
Africa	13 (4.5%)	7 (2.5%)	20 (3.5%)
Americas	1 (0.3%)	3 (1.1%)	4 (0.7%)
Asia	110 (38.1%)	113 (39.8%)	223 (38.9%)
Europe	95 (32.9%)	71 (25.0%)	166 (29.0%)
Oceania	3 (1.0%)	4 (1.4%)	7 (1.2%)
Australia and New Zealand	58 (20.1%)	73 (25.7%)	131 (22.9%)
Aboriginal and Torres Strait Islander	0 (0.0%)	1 (0.4%)	1 (0.2%)
Unknown	9 (3.1%)	12 (4.2%)	21 (3.7%)

Data are presented as *N*, mean (standard deviation), or *n* (percent).

### Primary Outcomes

The intervention group’s mean weight change was −0.23 kg (95% CI −0.89, 0.43) compared with +0.72 kg (95% CI 0.09, 1.35) in the usual care group (change difference −0.95 kg, 95% CI −1.87, −0.04; group by treatment interaction *p* = 0.04) over 12 mo. The intervention group’s mean change in waist circumference was −2.24 cm (95% CI −3.01, −1.42) compared with −1.74 cm (95% CI −2.52, −0.96) in the usual care group (change difference −0.50 cm, 95% CI −1.63, 0.63; group by treatment interaction *p* = 0.389) over 12 mo. The intervention group’s mean increase in fasting blood glucose was 0.18 mmol/l (95% CI 0.11, 0.24) compared with an increase of 0.22 mmol/l (95% CI 0.16, 0.29) in the usual care group (change difference −0.05 mmol/l, 95% CI −0.14, 0.05; group by treatment interaction *p* = 0.331) over 12 mo. Tables 2 and 3 show the results for the ITT analysis set for the 12-mo data; no other statistically significant results were identified across the primary and secondary endpoints when using the *F*-test of the group by time interaction—a result that was consistent for both the ITT and PPS analyses.

**Table 2:** Two-way table of predicted means (standard errors) and differences of means (*p*-values) for the co-primary endpoints of weight, waist circumference, and fasting blood glucose by treatment condition and time (intention-to-treat analysis).

Outcome	Control	Intervention	Difference
**Weight (kg) (*p* = 0.041)** ^**1**^			
Baseline^2^	74.61 (1.22)	76.67 (1.23)	2.05 (0.238)
12 mo^3^	75.34 (1.23)	76.44 (1.25)	1.10 (0.531)
Difference	0.72 (0.024)	−0.23 (0.498)	
**Waist (cm) (*p* = 0.389)** ^**1**^			
Baseline^4^	90.36 (0.87)	92.10 (0.87)	1.74 (0.158)
12 mo^5^	88.62 (0.88)	89.86 (0.90)	1.24 (0.326)
Difference	−1.74 (<0.001)	−2.24 (<0.001)	
**FPG (mmol/l) (*p* = 0.331)** ^**1**^			
Baseline^2^	4.72 (0.03)	4.82 (0.03)	0.10 (0.049)
12 mo^6^	4.94 (0.04)	4.99 (0.04)	0.05 (0.357)
Difference	0.22 (<0.001)	0.18 (<0.001)	

^1^ *p*-Value for the *F*-test of the time by treatment interaction.

^2^Control *n* = 289, intervention *n* = 284.

^3^Control *n* = 228, intervention *n* = 206.

^4^Control *n* = 289, intervention *n* = 283.

^5^Control *n* = 227, intervention *n* = 206.

^6^Control *n* = 227, intervention *n* = 205.

**Table 3:** Two-way table of predicted means (standard errors) and differences of means (*p*-values) for the secondary endpoints of blood pressure, blood lipids, and depressive symptoms by treatment condition and time (intention-to-treat analysis).

Outcome	Control	Intervention	Difference
**OGTT 2-h glucose (mmol/l) (*p* = 0.605)** ^**1**^			
Baseline	5.55 (0.10)	5.53 (0.10)	−0.02 (0.910)
12 mo	5.64 (0.11)	5.54 (0.12)	−0.10 (0.518)
Difference	0.10 (0.408)	0.01 (0.939)	
**Systolic blood pressure (mm Hg) (*p* = 0.526)** ^**1**^			
Baseline	110.52 (0.70)	112.23 (0.71)	1.71 (0.087)
12 mo	111.55 (0.76)	112.61 (0.79)	1.05 (0.338)
Difference	1.03 (0.148)	0.38 (0.610)	
**Diastolic blood pressure (mm Hg) (*p* = 0.721)** ^**1**^			
Baseline	70.15 (0.56)	71.56 (0.57)	1.41 (0.080)
12 mo	71.62 (0.61)	72.74 (0.63)	1.12 (0.203)
Difference	1.47 (0.008)	1.18 (0.042)	
**Total cholesterol (mmol/l) (*p* = 0.427)** ^**1**^			
Baseline	5.14 (0.06)	5.06 (0.06)	−0.08 (0.335)
12 mo	4.87 (0.06)	4.74 (0.06)	−0.13 (0.124)
Difference	−0.27 (<0.001)	−0.32 (<0.001)	
**Triglycerides (mmol/l) (*p* = 0.379)** ^**1**^			
Baseline	1.16 (0.04)	1.24 (0.04)	0.08 (0.140)
12 mo	1.22 (0.04)	1.26 (0.04)	0.04 (0.550)
Difference	0.06 (0.069)	0.02 (0.595)	
**LDL-C (mmol/l) (*p* = 0.417)** ^**1**^			
Baseline	3.07 (0.05)	3.01 (0.05)	−0.07 (0.341)
12 mo	2.92 (0.05)	2.81 (0.05)	−0.12 (0.126)
Difference	−0.15 (<0.001)	−0.20 (<0.001)	
**HDL-C (mmol/l) (*p* = 0.217)** ^**1**^			
Baseline	1.54 (0.02)	1.47 (0.02)	−0.07 (0.030)
12 mo	1.40 (0.02)	1.36 (0.02)	−0.04 (0.278)
Difference	−0.14 (<0.001)	−0.11 (<0.001)	
**Depressive symptoms (PHQ-9) (*p* = 0.132)** ^**1**^			
Baseline	4.57 (0.23)	4.06 (0.23)	−0.51 (0.111)
12 mo	4.39 (0.25)	4.41 (0.26)	0.03 (0.943)
Difference	−0.19 (0.449)	0.35 (0.172)	

^1^ *p*-Value for the *F*-test of the time by treatment interaction.

### Intervention Group Changes

Compared with baseline levels, the between-time comparisons at 3 mo show that mean weight change in the intervention group was −0.92 kg (*p* = 0.001) (Table 4). Other significant results in the intervention arm at 3 mo were a reduction in waist circumference, total cholesterol, HDL-C, and LDL-C (all *p <* 0.001). FPG was significantly higher at 3 mo than at baseline (*p <* 0.001) (Table 4). Reductions in waist circumference, total cholesterol, HDL-C, and LDL-C were maintained at 12 mo but not the reduction in weight. The increase in FPG persisted at 12 mo.

**Table 4:** Predicted means (standard errors) of primary and secondary endpoints for participants in the intervention group at baseline, 3 mo, and 12 mo.

Outcome^1^	Baseline	3 mo	12 mo	Baseline versus 3 mo	Baseline versus 12 mo	3 mo versus 12 mo
Weight (kg) (*p* = 0.002)	76.67 (1.22)	75.75 (1.23)	76.49 (1.23)	0.001	0.522	0.010
Waist (cm) (*p <* 0.001)	92.10 (0.89)	89.71 (0.91)	90.00 (0.91)	<0.001	<0.001	0.435
FPG (mmol/l) (*p <* 0.001)	4.82 (0.03)	4.97 (0.04)	5.00 (0.04)	<0.001	<0.001	0.513
Total cholesterol (mmol/l) (*p <* 0.001)	5.06 (0.05)	4.79 (0.06)	4.74 (0.06)	<0.001	<0.001	0.257
Triglycerides (mmol/l) (*p* = 0.771)	1.24 (0.04)	1.26 (0.04)	1.26 (0.04)	0.532	0.554	0.971
LDL-C (mmol/l) (*p <* 0.001)	3.01 (0.05)	2.83 (0.05)	2.81 (0.05)	<0.001	<0.001	0.538
HDL-C (mmol/l) (*p <* 0.001)	1.47 (0.02)	1.40 (0.02)	1.37 (0.02)	<0.001	<0.001	0.033

^1^ *p*-Value for the *F*-test of the time main effect.

### Lifestyle Modification Goals

Analysis of the proportion of participants meeting the MAGDA-DPP lifestyle modification goals adopted from the FIN-DPS did not reveal any significant time by group interactions. Similarly, there was no significant difference in the total number of goals achieved between the two groups (Table 5). S2 Fig. displays the association between average weight loss and different levels of engagement within the active intervention period (first 3 mo) and at 12 mo.

**Table 5:** Proportion of participants meeting the lifestyle modification goals in the MAGDA-DPP study at baseline and 12 mo, and total number of goals achieved at 12 mo (intention-to-treat analysis).

Lifestyle Modification Goal	Control	Intervention	*p*-Value
**≤30% of energy from fat**			
Baseline	23/287 (8.01%)	22/281 (7.83%)	0.966
12 mo	20/221 (9.05%)	25/204 (12.25%)	0.292
*p*-Value	0.544	0.070	0.366^1^
**≤10% of energy from saturated fat**			
Baseline	14/287 (4.88%)	17/281 (6.05%)	0.538
12 mo	12/221 (5.43%)	16/204 (7.84%)	0.264
*p*-Value	0.912	0.416	0.636^1^
**≥15 g/1,000 kcal fibre intake**			
Baseline	31/287 (10.80%)	33/281 (11.74%)	0.752
12 mo	28/221 (12.67%)	33/204 (16.18%)	0.249
*p*-Value	0.567	0.102	0.467^1^
**≥30 min/d moderate intensity physical activity**			
Baseline	114/289 (39.45%)	96/284 (33.80%)	0.146
12 mo	96/222 (43.24%)	90/198 (45.45%)	0.551
*p*-Value	0.498	0.005	0.108^1^
**≥5% reduction in body weight at 12 mo**	43/228 (18.86%)	43/206 (20.87%)	0.599
**Total number of goals achieved at 12 mo (missing excluded)** ^**2**^			
*N*	219	197	
Number of goals	0.89	1.02	0.201
**Total number of goals achieved at 12 mo (missing replaced)** ^**3**^			
*N*	289	284	
Number of goals	0.69	0.73	0.612

Data presented as *n/N* (percent) unless otherwise indicated.

^1^ *p*-Value for the Wald chi-squared test of the time by group interaction.

^2^Participants with one or more missing data points excluded.

^3^Missing data replaced with non-achievement of goal.

### Program PIPE and Process Evaluation

The MAGDA-DPP intervention was delivered via an RCT and did not have a specific target population for which penetration could be exactly calculated due to different recruitment streams. Some idea of penetration can be estimated from the NDSS mail-out invitation to participate, which was sent to 5,349 women registered with the NGDR and living in the study’s geographical catchment areas. Only 191 women responded to the NGDR invitation, and 149 of those subsequently agreed to participate in the intervention, resulting in a penetration rate of 4%, which is low. The implementation metric for MAGDA-DPP had a low level for frequency of sessions (32% of the number delivered within the US-DPP), a moderate level for duration (6–12 mo), and a high level for theoretical fidelity (standard curriculum and quality assurance measures implemented). The participation metric, based on enrolment of invited individuals, was low (26%) and reflects the challenge of engaging women in the intervention. The measures for PIPE effectiveness were all low: proportion of successful participants, average weight loss, and diabetes risk reduction (indirect, assessed against achievement of the five lifestyle modification goals adopted from the FIN-DPS, which are inversely associated with diabetes incidence [5]).

Among those randomised to the intervention (*n* = 284), 66% (*n* = 188) completed at least the individual session; specifically, 53% met the program minimum exposure definition of completing the individual session and one or more group sessions (*n* = 149), 34% had no exposure to the intervention (*n* = 96), 13% completed only the individual session (*n* = 37), and only 10% completed the individual session and all five group sessions (*n* = 28). Pregnancy rates and subsequent ineligibility for those completing the minimum intervention (11%, 16/149) and non-completers (14%, 16/135) were similar (*p* = 0.852). Group facilitators spent an average of 18 min per participant arranging intervention sessions and reminding participants. Despite an average of four attempts at contact made by facilitators via phone call or text message, 31% of women failed to attend a single session. To achieve the minimum intervention exposure (1 individual session and ≥1 group session), facilitators made on average ten contacts (mean total duration 20 min). Group facilitators made on average three contacts (mean total duration 8 min) to ensure attendance at a single session.

## Discussion

This study of a postnatal lifestyle intervention in women with gestational diabetes achieved a 1-kg weight difference compared with the control group. This difference is potentially significant for diabetes prevention, but the participation rate was low, reflecting how difficult it was to engage women in this cohort in the first year after the birth of their child. We found that, on average, women randomised to the MAGDA-DPP intervention group showed no postnatal weight gain, in contrast to women in the usual care group, who continued to gain weight over the 12-mo study period. The changes over 12 mo in the other two primary outcomes, the diabetes risk measures fasting blood glucose and waist circumference, were not significantly different for women in the intervention versus the control group. Intervention participants did show initial significant weight loss and improvements in their waist circumference and fasting blood glucose following the intensive component of the intervention, but these benefits were for the most part lost at 12 mo. This phenomenon is common amongst lifestyle modification programs and is a well-noted challenge in diabetes prevention in general [25,38,39] and in this population in specific [7,25,40,41]. Recruiting and delivering an intervention within this population of women with young families proved challenging, and our outcomes are similar to those recently reported by other studies [22,25,41].

Obesity is one of the strongest modifiable risk factors for T2DM development [5,42], and postnatal weight gain is a key risk factor for women [16,43], especially women with previous GDM [12,44]. Australian women typically gain 650 g annually [45], and the women in the MAGDA-DPP usual care group were no different (720 g average). The US Agency for Healthcare Research and Quality recently identified a 0.5-kg between-group weight gain difference as significant [46], and similarly the US Community Preventive Services Task Force found that even low levels of weight loss are effective in reducing T2DM risk [47]. Clinical significance has been attributed to a ~1-kg weight difference over time for cardiovascular disease [48] and T2DM [43], amongst other diseases. Wang and colleagues modelled a similar weight change within the US population and estimated that 2 million diabetes cases could be avoided with this small change [49]. Given that postnatal weight retention increases diabetes risk [11] and that guidelines recommend postnatal weight management [31,50,51], our findings could be interpreted as supporting the potential for a low intensity program to address postnatal weight retention and therefore lower diabetes risk. We would argue that our findings represent an issue of low penetration and participation in this target group, resulting in low effectiveness.

The number of reported DPPs specifically designed for women with prior GDM has risen exponentially, but their effectiveness in reducing diabetes risk has been low to date. A recent meta-analysis [41] found that no significant reduction in fasting blood glucose or any significant impact on weight loss occurred in DPPs of ≤6 mo duration. When the analysis was expanded to interventions of 12 mo duration, a significant difference in weight change of −1.06 kg (95% CI −1.68, −0.44) was seen, but this was driven by a single interim results publication for a study whose full intervention results have not been published [20]. It is clear that for effective weight loss within DPPs, high session frequency and longer program duration and fidelity are needed [37]. This presents a challenge for women with young families, who commonly cite a lack of time as a major barrier to engagement [17]. Nevertheless, a lower frequency of sessions can be effective for diabetes prevention—when delivered over longer periods of time and where penetration and participation rates are higher [37,47]—which is important when looking to sustainability or scaling up a program for health service delivery.

Central to the issue of penetration and participation is the design of randomised trials, which leads to the recruitment of highly selective populations. One of the largest DPPs in women with previous GDM comes from a study by Ratner and colleagues [7]; systematic reviews consistently [41,52,53] identify this study as high-quality evidence for the role of a DPP in this population, but the generalisability of the results from the population recruited is rarely discussed. Participants (*n* = 350) were mothers with IGT who were on average 43 y old, obese (with a mean BMI of 34.2 kg/m^2^), and with 12 y since their index GDM pregnancy. Clearly, their diabetes risk was higher, their child care demands lower, and the chance of engagement greater. In contrast, MAGDA-DPP mothers were 10 y younger, with BMI averaging in the overweight range, and only 2% had IGT. It is to be expected that their diabetes risk and their risk perception were likely to be quite different from those of Ratner et al.’s participants [7]. The recently published GEM trial [25] provides us with a more real-world perspective on the comparative effectiveness at the health service level. The population of the GEM trial (*n* = 2,280) was similar to that of MAGDA-DPP, but the trial’s penetration and participation was high as a result of the intervention being embedded as usual care within 22 randomised medical facilities and using telephone health coaching. The trial’s 12-mo weight loss outcomes showed significantly less postpartum weight retention in the intervention participants and a −0.64 kg weight difference between the intervention and control groups (95% CI −1.13, −0.14), lending support to our findings being more in line with real-world outcomes.

There are some lessons to be learnt from the factors contributing to the low effect size seen. The relatively low intervention engagement in MAGDA-DPP is reflected in an accordingly low level of behavioural change and resulting weight change. Attending and completing weight loss interventions are known correlates to achieving weight loss [54,55]; when people leave a program early, their skills and coping strategies for achieving and sustaining weight loss are likely to be underdeveloped [56,57]. Risk perception is another important influence on engagement with lifestyle behaviour change [36]. At the individual session, a risk algorithm was used to demonstrate the risk of developing diabetes to participants. Risk algorithms are highly age-dependent; most women were normoglycemic, so it is possible their interpretation was that they did not need to worry about their risk of diabetes until they were older.

Strengths of this randomised trial include the length of follow-up after the active intervention, good retention rates, the fidelity measures included in the intervention design, and the rigorous data collection methodology. Limitations of the MAGDA-DPP study include the low level of participation in the intervention group sessions along with overall low levels of penetration and participation, as defined by the PIPE metric [37]. Although relatively extensive consultation work was undertaken prior to MAGDA-DPP implementation (literature review, qualitative interviews with the population of interest [18], piloting of the program materials in postnatal women who had gestational diabetes), it is possible that a broader qualitative exploration of issues relating to penetration, compliance, and program delivery may have yielded stronger engagement and possibly better outcomes. The diabetes risk profiles for MAGDA-DPP participants were surprisingly low considering the body of evidence behind GDM being a strong risk factor for T2DM development [3,11]. At baseline, 10% of MAGDA-DPP participants were identified as having prediabetes, and their average BMI was only 1 kg/m^2^ higher than the average Australian woman [58]. It is also possible that those who agreed to participate were a lower-risk group, with healthier baseline behaviours. Another possible study limitation was the study protocol specification of three co-primary endpoints without a plan to test each at a stringent significance level, or in a hierarchical manner, in order to maintain a trial-wise type I error rate below, say, the conventional 5%. Our protocol [30] did state that a “statistically significant change in any one of these three endpoints will be regarded as evidence of a change in diabetes risk”, and we found a statistically significant difference between the groups for weight change. The observed magnitude of the difference is similar to the magnitudes reported in other studies of lifestyle interventions [25,41], and we believe it is important to add the result of this study to the accumulating knowledge about the utility of lifestyle modification programs in mothers with prior GDM.

### Translation in Policy and Practice

Our trial explored the effect of offering a DPP in the first year postnatally and showed that it was ineffective. Telephone- or web-based interventions that can adapt to the time demands of raising a young family may have more successful participation rates [23,25] and may have the advantage of being less resource intensive and more suited to scale-up, but it is unlikely that they will be as effective as programs offered to women with the high-risk characteristics of those in the study by Ratner et al. [7].

The extent to which the newer GDM diagnostic criteria of the International Association of Diabetes and Pregnancy Study Groups will affect demand for diabetes prevention services in not yet known [59], but our finding that the majority of our cohort were at low risk (using the previous, higher GDM diagnostic cut-offs) suggests that the relative benefit and cost associated with offering an early postnatal period DPP to all women with a previous GDM pregnancy does not make it a sensible use of scarce health resources. A better health service approach might be to improve the currently recommended annual diabetes screening within family medicine practice for women with previous GDM, so more women with prediabetes, who are at high risk, can be identified [50]. This health service approach could be supported by a reminder system within a national GDM registry, the NGDR being the current Australian example, and women with prediabetes could be more selectively targeted for recruitment into an appropriate DPP.

### Conclusions

Our results show that a low intensity, group-delivered DPP was superior to usual care in preventing postnatal weight gain in a cohort of women with previous GDM. However, the level of engagement was low, and DPPs may need to be offered at other time points after pregnancy. Further research on engagement is required, including participant input into the design of interventions, and a more effective option may be to follow up women with previous GDM until they show IGT or HbA1c levels in the prediabetes range before offering entry to a DPP.

## Supporting Information

S1 Text Trial protocol. (PDF)

S2 Text CONSORT statement. (DOCX)

S3 Text Trial protocol amendment. (PDF)

S4 Text Statistical analysis plan. (PDF)

S1 Fig Trial flowchart for intervention format and testing activity. (TIF)

S2 Fig Average weight change following completion of the active intervention (3-mo time point) and at intervention completion (12-mo time point), split by session attendance. Minimum to moderate engagement was defined as attending the individual session and 1–4 group sessions; full engagement was attending all sessions.
(TIF)

## Abbreviations

DPP: diabetes prevention program
FIN-DPS: Finnish Diabetes Prevention Study
FPG: fasting plasma glucose
GDM: gestational diabetes mellitus
GGT-DPP: Greater Green Triangle Diabetes Prevention Program
HDL-C: high-density lipoprotein cholesterol
IGT: impaired glucose tolerance
ITT: intention-to-treat
LDL-C: low-density lipoprotein cholesterol
MAGDA-DPP: Mothers After Gestational Diabetes in Australia Diabetes Prevention Program
NDSS: National Diabetes Services Scheme
NGDR: National Gestational Diabetes Register
OGTT: oral glucose tolerance test
PHQ-9: Patient Health Questionnaire 9
PIPE: penetration, implementation, participation, and effectiveness
PPS: per protocol set
RCT: randomised controlled trial
T2DM: type 2 diabetes mellitus
US-DPP: US Diabetes Prevention Program
