# Supplementary material for: Mothers after Gestational Diabetes in Australia (MAGDA): A Randomised Controlled Trial of a Postnatal Diabetes Prevention Program
Source: PLoS Med. 2016 Jul 26;13(7):e1002092. doi: 10.1371/journal.pmed.1002092 (PMC4961439; doi:10.1371/journal.pmed.1002092)
Supplement: S1 Text — (PDF) [file pmed.1002092.s001.pdf]

Shih *et al. Trials* 2013, **14**:339
http://www.trialsjournal.com/content/14/1/339

TRIALS

STUDY PROTOCOL Open Access

Mothers After Gestational Diabetes in Australia Diabetes Prevention Program (MAGDA-DPP) post-natal intervention: study protocol for a randomized controlled trial

Sophy TF Shih[1], Nathalie Davis-Lameloise[2], Edward D Janus[2,3], Carol Wildey[2,4], Vincent L Versace[2], Virginia Hagger[5], Dino Asproloupos[2,4], Sharleen O'Reilly[6], Paddy A Phillips[7], Michael Ackland[8], Timothy Skinner[9], Jeremy Oats[10], Rob Carter[1], James D Best[11], James A Dunbar[2*] and On behalf of the MAGDA Research Group

Abstract

Background: Gestational diabetes mellitus (GDM) is defined as glucose intolerance with its onset or first recognition during pregnancy. Post-GDM women have a life-time risk exceeding 70% of developing type 2 diabetes mellitus (T2DM). Lifestyle modifications reduce the incidence of T2DM by up to 58% for high-risk individuals.

Methods/Design: The Mothers After Gestational Diabetes in Australia Diabetes Prevention Program (MAGDA-DPP) is a randomized controlled trial aiming to assess the effectiveness of a structured diabetes prevention intervention for post-GDM women. This trial has an intervention group participating in a diabetes prevention program (DPP), and a control group receiving usual care from their general practitioners during the same time period. The 12-month intervention comprises an individual session followed by five group sessions at two-week intervals, and two follow-up telephone calls. A total of 574 women will be recruited, with 287 in each arm. The women will undergo blood tests, anthropometric measurements, and self-reported health status, diet, physical activity, quality of life, depression, risk perception and healthcare service usage, at baseline and 12 months. At completion, primary outcome (changes in diabetes risk) and secondary outcome (changes in psychosocial and quality of life measurements and in cardiovascular disease risk factors) will be assessed in both groups.

Discussion: This study aims to show whether MAGDA-DPP leads to a reduction in diabetes risk for post-GDM women. The characteristics that predict intervention completion and improvement in clinical and behavioral measures will be useful for further development of DPPs for this population.

Trial registration: Australian New Zealand Clinical Trials Registry ANZCTRN 12610000338066

Keywords: Gestational diabetes, Post-natal, Lifestyle intervention, Type 2 diabetes prevention

Background

Type 2 diabetes (T2DM) and gestational diabetes mellitus (GDM) are escalating problems worldwide. Depending on the population studied, 1 to 14% of all pregnancies are complicated by GDM [1]. This estimate may double if the International Diabetes Association of Diabetes and Pregnancy Study Groups (IDAPSG) recommended criteria are implemented [2].

Gestational diabetes mellitus is defined as glucose intolerance of variable severity with onset or first recognition during pregnancy [3]. Post-GDM women have a life-time risk of developing T2DM exceeding 70% [4]. In Australia, between 2005 and 2006, GDM was diagnosed in 4.6% of hospital births, and the incidence increased by 22% over 6 years from 3.6 per 100 pregnancy in 2000/01 to 4.4 per 100 pregnancy in 2005/06 [5]. Risk factors for GDM include family history of diabetes, age

* Correspondence: director@greaterhealth.org
[2]Greater Green Triangle University Department of Rural Health, Flinders University, PO Box 423, Warrnambool VIC 3280, Australia
Full list of author information is available at the end of the article

BioMed Central

© 2013 Shih et al.; licensee BioMed Central Ltd. This is an open access article distributed under the terms of the Creative Commons Attribution License (http://creativecommons.org/licenses/by/2.0), which permits unrestricted use, distribution, and reproduction in any medium, provided the original work is properly cited.

over 30 years, overweight or obesity at time of conception, and belonging to a high-risk ethnic group (for example, Aboriginal and Torres Strait Islander, Pacific Islander, South Asian, Middle Eastern) [6,7].

Pregnancies complicated by GDM have increased incidence of fetal macrosomia. This in turn leads to an increased risk of caesarean deliveries, maternal preeclampsia, and neonatal complications including hypoglycemia, shoulder dystocia, and birth trauma [7]. Women with GDM also have a significantly increased risk (by approximately 25% of absolute risk) of developing T2DM within 15 years relative to women without GDM [8] and a 30 to 69% increased risk of developing GDM in subsequent pregnancies [9]. GDM also puts the child at risk, with the offspring of mothers with GDM having an increased risk of obesity and abnormal glucose metabolism during childhood, adolescence, and adulthood [10].

In Australia, the prevalence of T2DM has more than doubled since 1981, with 3.8% of Australians, almost one million, having been diagnosed with T2DM in 2007 to 2008 [11]. It is also estimated that at least half of those who have T2DM are undiagnosed, and so are unaware of their condition [12]. Currently, diagnosed diabetes is the second highest contributor to the Australian burden of disease, responsible for 5.2% of disability adjusted life-years. It is estimated that by 2023, T2DM will be the leading contributor, responsible for 8.6% of overall disease burden [13], resulting from the major morbidity of diabetes complications. Diabetes also poses an enormous economic burden, accounting for A$1.4 billion (or US$ 1.26 billion, approximate exchange rate A $1 to US$0.9) in healthcare expenditure in 2003, which is projected to increase to almost A$7 billion (US$6.3 billion) by 2033 [14].

Therefore, there is an urgent need to implement a widespread and coordinated approach to prevent T2DM. Several clinical trials have shown that lifestyle modification with weight loss and moderate exercise can reduce the incidence of T2DM by up to 58% for people at high risk [15-17]. Indeed, these lifestyle modification programs have been shown to be even more effective than drug treatment in clinical trials [7,16,18], and have a lasting effect that is still evident eight years from the onset of intervention and four years after the active intervention has ceased. The diabetes prevention program conducted in the United States (US DPP) included 350 women with previous GDM [7]. The results showed a greater conversion to diabetes for GDM than non-GDM women; however, both groups responded to metformin treatment and lifestyle interventions [7].

One way to approach this problem is target individuals at high risk of developing diabetes. Previous studies such as the US DPP and the Finnish prevention study [19] have targeted individuals with glucose dysregulation as identified by an oral glucose tolerance test. A history of GDM is one of the major risk factors in women who should be targeted for preventing development of T2DM.

Mothers After Gestational Diabetes in Australia study

The Mothers After Gestational Diabetes in Australia (MAGDA) aims to develop and implement a macro-level system change to reduce the risk of progression to T2DM for women with previous GDM. The project consists of four components: (1) a register to facilitate appropriate follow-up after diagnosis of GDM; (2) an intervention to reduce progression to T2DM; (3) a health economics evaluation of the register and intervention; and (4) an understanding of how to implement the register and follow-up in general practice. This paper presents the protocol for the intervention, a randomized controlled trial (RCT) of the Mothers After Gestational Diabetes in Australia Diabetes Prevention Program (MAGDA-DPP). The MAGDA-DPP trial offers an evidence-based structured lifestyle modification group-based intervention for women who have had GDM. The objectives of MAGDA-DPP are that the intervention will result in favorable changes, relative to usual care, in clinical, behavioral and patient-relevant outcome. In addition, MAGDA-DPP aims to identify individual characteristics predictive of successful outcome.

Methods/Design

Study design

MAGDA-DPP is a prospective, open RCT to assess the effectiveness of a structured diabetes prevention program (DPP) for women who have had GDM. This trial has an intervention group participating in a DPP and a control group receiving usual care from their general practitioners (GPs) during the same time period. Women will be recruited from two Australian State capital cities, Melbourne and Adelaide.

All women recruited into the study will be followed up for 12 months. At baseline, the women will undergo an assessment consisting of blood tests, anthropometric measurements, and self-reported assessment of health status, diet, physical activity, quality of life, depression and risk perception. The intervention group will be assessed at baseline, and at three and 12 months. The control group will be assessed at baseline and 12 months.

The MAGDA-DPP intervention is coordinated by Deakin University (Melbourne Campus) and is supported by a 'Partnerships for Better Health' Grant from the National Health and Medical Research Council of Australia (NHMRC). The project includes multiple study partners, consisting of two state governments, three universities and two non-government organizations. Study

partners, recruiting hospitals and relevant ethics approvals are listed in Table 1.

Selection of participants

The inclusion criterion for women is diagnosis of GDM in their most recent pregnancy, with GDM defined by the Australasian Diabetes in Pregnancy Society (ADIPS) criteria: fasting plasma glucose (FPG) of 5.5 mmol/L or higher, and/or 2 hour glucose of 8.0 mmol/L or higher on a 75 g oral glucose tolerance test (OGTT) [3], or a glucose challenge test (GCT) result of 12.0 mmol/L or higher.

The exclusion criteria are: (1) pre-existing diabetes (Type 1 or 2 diabetes mellitus); (2) cancer (not in remission); (3) severe mental illness; (4) substance abuse (illicit drugs); (5) myocardial infarction in the preceding three months; (6) difficulty with English; (7) involvement in another post-natal intervention trial; and (8) pregnancy at post-natal baseline testing or at any point during the 12 months of study involvement.

Once women diagnosed with GDM express interest in the study, the recruiter (the MAGDA-DPP project manager or research assistant) will conduct the first eligibility screening, generally prior to the women delivering their babies. A subsequent post-natal screening will be conducted at time of baseline OGTT, to exclude women who have diabetes at that stage.

Recruitment process

Multiple recruitment strategies will be used for MAGDA-DPP. The study will be promoted by mailed leaflets via the National Diabetes Services Scheme (NDSS) (using data from the National GDM Register) [20] to women living in relevant postcodes in Adelaide (South Australia) and Melbourne (Victoria). Women will be recruited predominantly through participating hospitals, referrals from private healthcare providers and retrospective database mining of hospital records. Other specific recruitment activities may be used in consultation with the diabetes education service at each hospital. Women diagnosed with GDM will be informed about the study by diabetes educators, either individually or in a group.

Prospective recruitment will take place at The Royal Women's Hospital and Sunshine Hospital in Melbourne and Lyell McEwin Hospital in Adelaide. Because of late participation in the study by Women's and Children's Hospital and Flinders Medical Centre in Adelaide, retrospective as well as prospective recruitment will take place at these hospitals only, allowing women diagnosed with GDM after July 1, 2011 to participate in the study (Table 1).

At the hospital recruitment sites, women newly diagnosed with GDM will be referred, if they agree, to the MAGDA recruiter. Alternatively, potential participants will be identified by their medical history or from the list of newly diagnosed patients, with the assistance of the clinic coordinator. Women will be approached by the recruiter in the antenatal clinic waiting room or when attending diabetes education group sessions. Patients will receive a study outline and will be offered participation in the study. At the hospital site's group diabetes

Table 1 MAGDA project study partners, recruitment sites and relevant ethics approvals

Study partner/recruitment site (state)	Ethics approval authorities (reference number)
Deakin University (VIC)	Deakin University human research ethics committee (2010–005)[1]
Flinders University (SA)	Southern Adelaide Clinical Network human research ethics committee (330/10)
The University of Melbourne (VIC)	NA
Department of Health (VIC)	NA
South Australia Health (SA)	South Australia Health human research ethics committee (339/02/2013)
Diabetes Australia Victoria branch (VIC)/NDSS	Deakin University human research ethics committee (2010–005)[1]
GP-Victoria	NA
The Royal Women's Hospital (Vic)	The Royal Women's Hospital (10/06)
Sunshine Hospital (VIC)	Melbourne Health (2010.008)
Lyell McEwin Hospital (SA)	The Queen Elizabeth Hospital/Lyell McEwin Hospital HREC (2011109)[2]; South Australia Health human research ethics committee (339/02/2013); South Australia Aboriginal Health research ethics committee (04-10-331)
Flinders Medical Centre (SA)	South Australia Health human research ethics committee (339/02/2013); South Australia Aboriginal Health research ethics committee (04-10-331); Southern Adelaide Clinical Network human research ethics committee (330/10); Southern Adelaide Health Service (SSA/12/SAC/170)
Women's and Children's Hospital (SA)	South Australia Health human research ethics committee (339/02/2013); South Australia Aboriginal Health research ethics committee (04-10-331); Women's and Children's Hospital (SSA/13/WCHN/73)

Abbreviations: NA not applicable, *NDSS* National Diabetes Service Scheme, *SA* South Australia, *VIC* Victoria.
[1]Lead human research ethics committee for the overall project.
[2]Lead human research ethics committee in South Australia.

education sessions, the recruiter will discuss the risk of progression to T2DM and outline the study. Eligibility screening will be carried out for all interested women, and consent forms will be provided at the session or mailed after the session. If a woman is not interested in participating at first approach or withdraws at any other stage of the study, the reasons for her decision will be sought.

Consent

Eligible women will be given a Participant Information and Consent Form (PI&CF) to review with the recruiter. If there is inadequate time to review the PI&CF, a copy will either be provided directly to the participants or mailed to them along with a prepaid envelope in which to return the completed consent form. If the consent form is not received within four weeks, the recruiter will make contact with the woman. At three months postpartum for all live births, the MAGDA-DPP recruiter will follow up with a phone call to verify if an OGTT at six to eight weeks postpartum has been performed. At three months postpartum and onwards, if the woman does not have diabetes, the study nurse/phlebotomist will make contact to organize for baseline data collection. Once the results of the baseline blood tests are known, women with confirmed diabetes will be informed, referred to their nominated GP and excluded from the study. If they do not have diabetes, they will be randomized into either the control or the intervention group. The recruitment flowchart is shown in Figure 1.

Randomization

Block randomization will be implemented and separate randomization lists for each 'venue' or 'stratum' will be

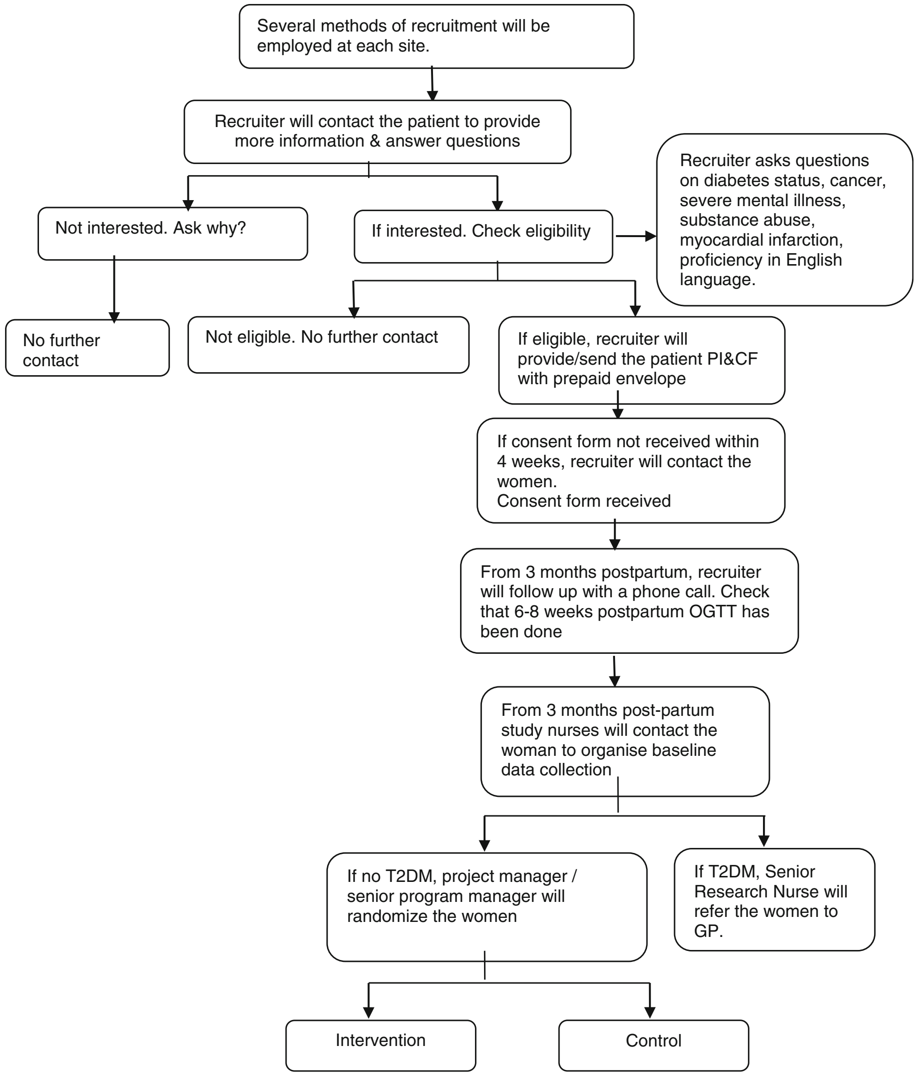

Figure 1 Recruitment flowchart.

created. Each list will be supplied as a separate file and will be imported into the current MAGDA management database, which selects the next available sequence number for the venue/stratum. This sequence number will be displayed, along with the assignment code (control or intervention) to the user in the randomization office at Deakin University.

Women will be randomized into either the intervention or control arm after the baseline blood results have been received by the MAGDA-DPP project manager. If the woman does not have diabetes, the project manager or senior program manager will randomize her using the MAGDA-DPP management database. Each woman will receive a letter with the outcome of her randomization along with her baseline test results, and a copy will be sent to her nominated GP.

Intervention

The theoretical framework of the MAGDA-DPP intervention is based on the Health Action Process Approach (HAPA), and supported by social cognitive theory [21,22] and self-regulation theory [23-25], which has informed the structuring of the program and activities in each session. The intervention was based on the *Life!* program [26], which was previously shown to be effective in affecting change in diabetes risk factors. The theory driven components of the program were enhanced and the content of sessions tailored to reflect the issues that previous work has shown to be of relevance to this population (for example, sleep deprivation, post-natal depression). The intervention delivery is driven by a facilitator's manual that outlines the core content and behavioral change processes to be used in each session of the program. Fidelity of the program will be established by assessment of the audio recordings when the facilitator is delivering the sessions.

The intervention group will be offered a series of six face-to-face sessions delivered by trained healthcare professionals, with two additional follow-up telephone calls at a later stage. Table 2 summarizes the core curriculum of the sessions. The first session will be an individual session carried out in the woman's home, nominated community health center or Aboriginal health center. The initial session will be followed by five group sessions held at two week intervals and two subsequent phone calls at three and six months after the final group session. Each group session will be two hours long with up to 15 women per group. The control group will continue with usual care and be offered the intervention program after 12 months subject to timing or availability of MAGDA-DPP courses. An indicative timeline from recruitment to completion of the follow-up is illustrated in Figure 2.

At the first individual session, the participant will be provided with an intervention program handbook. The intervention program aims to encourage participants to achieve the following five goals: (1) reduce fat intake (no more than 30% of energy from fat); (2) decrease saturated fat intake (no more than 10% of energy from saturated fat); (3) increase fiber intake (at least 15 g per 1000 kcal); (4) increase physical activity (at least 30 minutes of moderate exercise per day); and (5) reduce body weight (at least 5% of body weight reduction within 12 months).

Outcome assessment

At baseline and completion of the study (12 months after baseline), primary (changes in diabetes risk) and secondary (changes in cardiovascular disease risk factors, psychosocial and quality of life measurements) outcome will be assessed.

Primary outcome

The primary outcome is the change in diabetes risk, as determined by changes in FPG, weight, or waist circumference. A statistically significant change in any one of these three endpoints will be regarded as evidence of a change in diabetes risk. No adjustments to significance levels will be made for multiple testing. These co-primary outcome will be assessed at baseline and 12 months for all women, and additionally at 3 months (or as soon as possible after the final group session) for the intervention participants only.

Secondary outcomes

Changes in glucose (2 hour OGTT), fasting lipids, blood pressure, depression, quality of life, physical activity and diet are the secondary outcome, which will be assessed at baseline and 12 months for all women.

Anthropometric measurements and laboratory testing

To ensure that the clinical measurements meet research standards, measurements will be carried out by specially trained study nurses/phlebotomist. All clinical tests will follow the latest international recommendations from the European Health Risk Monitoring protocol [27]. Blood samples will be collected by the study nurse/phlebotomist, and analyzed by Melbourne Pathology (Victoria) or ClinPath (South Australia). Anthropometric measurements including height, weight, waist and hip circumference, and blood pressure will be taken. Women will be asked to fast from 10 pm the night before their appointment and venous samples will be drawn at the appointment to analyze lipids (triglycerides, total cholesterol, low-density lipoprotein and high-density lipoprotein cholesterol), HbA1c and glucose (fasting and 2 hour OGTT). Additional blood from women who consent to having their blood stored will be bio-banked at −80°C for further additional analyses.

For the intervention group only, at three months after baseline testing or as soon as possible after the final

Table 2 MAGDA-DPP core curriculum and key contents of the six sessions and two follow-up telephone calls

Session	Topics covered
Individual session (delivered in participant's home at >3 months postpartum)	Introduction to DPP
	Assessment of personal risk of developing T2DM
	Highlight the five program goals:
	• Reduce weight by 5%
	• Reduce total fat intake to < 30% daily energy intake
	• Reduce saturated fat intake to < 10% daily energy intake
	• Increase fiber intake to > 15 g per 1000 kcal
	• Increase physical activity to at least 30 minutes moderate intensity physical activity on at least 5 days per week
	Build commitment to attend the program by discussed perceived benefits and importance of attending
	Establish personal weight goal (current weight minus 5%) for the after 3 months
	Set physical activity goal
Group session 1 (held in community venue within 1 month of individual session)	Background to the program
	What is diabetes?
	Physical activity goal-setting: review of progress with goal set at the individual session
	Fighting fat: saturated fat
	• Benefits of reducing saturated fat intake
	• Identify common sources of saturated fats in foods and from food labels
	• Practice modifying foods to reduce saturated fat content
	Saturated fat goal-setting
Group session 2 (held in community venue 2 weeks after Group session 1)	Review goals set, and set new activity goal
	Fighting fat: total fat
	• Benefits of reducing total fat intake
	• Identify sources of dietary fat
	• Self-assessment of current total fat intake
	• Practice modifying foods to reduce total fat content
	Weight management
	Total fat goal-setting
Group session 3 (held in community venue 2 weeks after Group session 2)	Review goals set, and set new activity goal
	Filling up on fiber
	• Benefits of increasing fiber intake
	• Goals for daily fiber intake
	• Self-assessment of current fiber intake
	• Identify high-fiber foods
	• Identify meal plans that are high in fiber
	Family healthy eating (part 1)
	• Healthier shopping activity
	Healthy eating goal-setting (1)
Group session 4 (held in community venue 2 weeks after Group session 3)	Review goals set, and set new activity goal
	Family healthy eating (part 2)
	• Healthier family meal-planning
	• Dealing with barriers to making healthier choices
	Mindful eating
	Managing sleep
	• Importance of good family sleep patterns
	• Ways to improve sleep
	Healthy eating goal-setting (2)
Group session 5 (held in community venue 2 weeks after Group session 4)	Review goals set and set new activity goal
	Stress
	• Impact of stress on feelings and behaviors and health
	• Identify sources of stress
	• Identify unhelpful stress-management strategies
	• Discuss more helpful ways of managing stress
	Depression
	• Identifying depression
	• Impact of depression
	• Identifying ways to manage depression
	• Resources available for support
	Relapse prevention
	• Weight and energy balance
	• Managing relapses
	• Key strategies for maintaining a healthier lifestyle
	Long-term goal-setting
	• Action-planning
	• Problem-solving
	• Rewards
Telephone follow-up 1 (3 months after Group session 5)	Review of progress
Telephone follow-up 2 (6 months after Group session 5)	Review of progress

group session, a follow-up assessment will repeat all blood tests (except OGTT), and anthropometric measurements of weight and waist circumference. All women who complete this clinical testing will receive a standardized

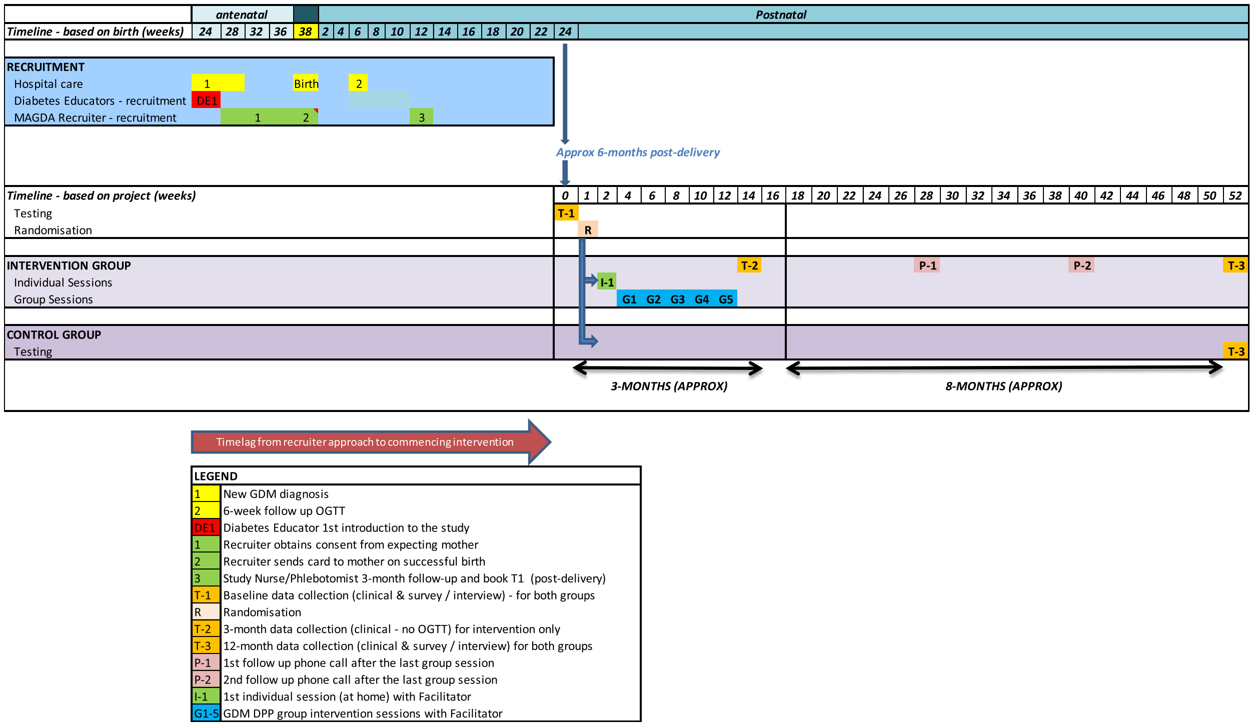

Figure 2 Indicative timeline from recruitment to completion of follow-up.

feedback letter documenting their results and any evidence of high blood pressure or depression. A copy will be sent to their nominated GP.

Self-report measures

Women will be asked to complete a comprehensive survey that includes demographic questions (at baseline only); measures of health behavior including the Food Frequency Questionnaire [28], the Active Australia Questionnaire [29], self-regulation [30] and self-efficacy for diet and physical activity [23,24,31,32]; social support (Multidimensional Scale of Perceived Social Support [33]); quality of life (Assessment of Quality of Life, AQoL-8D) [34]; depression and suicidal ideation (Patient Health Questionnaire 9, PHQ9 [35]); and health status questions (including demographics, smoking status, and history of diabetes, myocardial infarction, cancer, and mental disorders) at baseline and 12 months post-baseline.

If a woman scores 11 points or more on the PHQ9 and/or if she scores on the suicidal-ideation question (question 9), this result will be discussed with her at the time to establish whether she is aware of her depression risk, and she will be advised to attend her GP for further discussion and management. This will also be highlighted in the results feedback letter to the woman and her GP.

A healthcare resource use cost diary will be given to all women to record utilization of diabetes-related healthcare services during the time from baseline to completion of the study. The diary aims to collect costs associated with activities related to GDM follow-up (such as weight management and measurement of blood pressure, glucose and lipid), and will include healthcare, time, and travel costs. An additional purpose of the diary is to determine any differences between the intervention and control groups in seeking advice about lifestyle change and diabetes prevention.

Sample size and power calculation

For the estimation of sample size for two groups, using a two-sided 5% significance level and 80% power, the total number required will be 430 (215 in each arm). The sample size calculation was based on the observed mean change in FPG over 12 months in the Greater Green Triangle Diabetes Prevention Program (GGT DPP) study [36] and is powered to detect an effect size of 0.27 (assuming mean difference between intervention and control groups of 0.14 mmol/L and within group standard deviation of 0.5 mmol/L). To allow for an estimated attrition rate of up to 25% (estimate based on the GGT DPP), we will require a total sample of 574 (287 in each arm). This sample size will provide sufficient power to also analyze changes in weight and waist circumference. The intention-to-treat (ITT) principle will be adhered to, and sensitivity analysis will also be carried out.

Data collection

Participants' blood test results will be uploaded onto the Melbourne Pathology/ClinPath websites, which are password-encrypted. In order to protect confidentiality, the participant ID number and date of birth will be the only identifying information available to the laboratory. Melbourne Pathology will also post hard copies of the pathology results to the study office (Deakin University), and ClinPath will send their hard copy results to the study office in South Australia in secure envelopes via ClinPath couriers.

Web-based survey forms will be used primarily to collect information during the testing appointment, with the paper-based system used as a back-up. Clinical measurements will be recorded by the study nurse. All questionnaires are self-reported by the participating women, and will use the participant ID number (on the front of each form/questionnaire) as the identifier. Only the demographic questionnaire contains the participants' contact details, and this will be maintained separately from all of the other information relating to each woman.

Data storage and management

All data will be stored on Deakin University eSolutions servers (ISO 9000 compliant for security, access and quality control) and the National eResearch Collaboration Tools and Resources (NeCTAR) server. Data collected by the research team will be transferred to locked cabinets at the respective study offices, and 10% of hard copy questionnaires will be randomly audited for quality control.

Data analysis

Analyses will be performed using STATA version 12 or later (Stata Corp., College Station, TX, USA) and the ITT principle will be adhered to. Baseline characteristics will be compared between the intervention and control groups using χ^2 tests, independent t-tests, and/or the Wilcoxon rank-sum test, as appropriate. Primary and secondary outcome (as described above) will be evaluated using mixed models, treating 'group' as a between-subject factor, and 'time' as a within-subject factor. Two-sided tests will be used, with a level of $P<0.05$ determining statistical significance.

A separate and parallel economic evaluation protocol will be described elsewhere.

Discussion

Gestational diabetes mellitus is the strongest single population predictor of T2DM. At least 20 to 50% of women who have GDM will go on to develop diabetes within 28 years [8,37], and women with GDM have a life-time risk exceeding 70% of developing T2DM [4]. Lifestyle-modification programs are effective in delaying or preventing diabetes in the general high-risk population. Although there were some post-GDM women in the US DPP [7], no intervention program has previously been specifically designed and tested for this group.

This study is necessary because it is important to know if the MAGDA-DPP intervention leads to a reduction in diabetes risk in women with a history of GDM. Defining the participants' characteristics that predict attendance and completion of the intervention and improvement in clinical and behavioral measures will be useful for further development of DPPs for this population in Australia and internationally.

Trial status

Recruitment in Melbourne (Victoria) commenced at The Royal Women's Hospital in January 2011, followed by Sunshine Hospital in November 2011 and in Adelaide (South Australia) at Lyell McEwin Hospital in January 2012. By June 4, 2013, 1,517 women diagnosed with GDM have been approached during their clinic appointments at the above three hospitals. Of these, 340 women have given written consent, 219 have not yet returned their consent form, 701 have declined to participate in the study and 257 were ineligible. Recruitment, both retrospectively and prospectively, at Women's and Children's Hospital and Flinders Medical Centre in Adelaide is expected to commence in mid-2013.

Baseline testing commenced in July 2012 in Melbourne (Victoria) and March 2013 in Adelaide (South Australia). By June 4, 2013, 134 women had been randomized to either the intervention or the control group. The first MAGDA-DPP intervention group started in November 2012, and there have been six groups, four in Melbourne (Victoria) and two in Adelaide (South Australia), initiated since then.

Abbreviations

AQoL-8D: Assessment of quality of life; DPP: Diabetes prevention program; FPG: Fasting plasma glucose; GCT: Glucose challenge test; GDM: Gestational diabetes mellitus; GGT DPP: Greater green triangle diabetes prevention program; GP: General practitioner; HAPA: Health action process approach; IDAPSG: International diabetes association of diabetes and pregnancy study groups; MAGDA-DPP: Mothers after gestational diabetes in Australia diabetes prevention program; NDSS: National diabetes services scheme; NeCTAR: National eResearch collaboration tools and resources; NHMRC: National health and medical research council of Australia; OGTT: Oral glucose tolerance test; PHQ9: Patient health questionnaire; PI&CF: Participant information and consent form; SA: South Australia; T2DM: Type 2 diabetes mellitus; VIC: Victoria.

Competing interests

The authors declare that they have no competing interests.

Authors' contributions

EJ, PP, MA, JO, RC, JB, and JD developed the research question, designed the study, and were responsible for obtaining funding for the study. VH, TM, and SOR designed the intervention for the group work and its evaluation. SS wrote the first draft of this manuscript, and with NDL, EJ, and VV, was responsible for the revisions of the manuscript. DA and CW were responsible for the amendment of the study protocol, and contributed to specific

sections of the manuscript. VV is the statistician, and contributed to specific sections of randomization, sample and power calculation, and data analysis. JD is the guarantor and general supervisor of the study, and was involved in revising this article. All authors read and approved the final version of the manuscript.

Acknowledgements

The study was funded by a Partnerships for Better Health Grant from the National Health and Medical Research Council (grant number 533956) with financial and in-kind contributions from the Victoria State Government, South Australia State Government and Diabetes Australia Victoria. We thank Dr Rosalind Lau and Professor Prasuna Reddy for the initial development of the study protocol. We also thank Kate Schlicht, Associate Professor John Reynolds and Professor Catherine Bennett for their advice on aspects of psychology, statistics, and epidemiology, respectively. We also acknowledge the contributions of the MAGDA Manual Training Committee and MAGDA RCT Working Group to the intervention handbooks and the study protocol revision. The views expressed in this manuscript are those of the authors and do not necessarily represent the views of, nor should be attributed to, the National Health and Medical Research Council, Department of Health Victoria, South Australia Health and Diabetes Australia Victoria.

Author details

[1]Deakin Health Economics, Population Health Strategic Research Centre, Faculty of Health, Deakin University, 221 Burwood Highway, Burwood VIC 3125, Australia. [2]Greater Green Triangle University Department of Rural Health, Flinders University, PO Box 423, Warrnambool VIC 3280, Australia. [3]Department of Medicine, North West Academic Centre, The University of Melbourne, Western Centre for Health Research and Education, Western Health, 176 Furlong Rd, St Albans VIC 3021, Australia. [4]Deakin Health Services Implementation Research Unit, Population Health Strategic Research Centre, Faculty of Health, Deakin University, 221 Burwood Highway, Burwood VIC 3125, Australia. [5]Diabetes Australia – Victoria, Melbourne, 570 Elizabeth Street, Melbourne VIC 3000, Australia. [6]Centre for Physical Activity and Nutrition Research, Faculty of Health, Deakin University, 221 Burwood Highway, Burwood, VIC 3125, Australia. [7]South Australia Health, GPO Box 2100, Adelaide SA 5001, Australia. [8]Office of the Chief Health Officer, Department of Health, 14/50 Lonsdale Street, Melbourne, VIC 3000, Australia. [9]Psychological and Clinical Sciences, Charles Darwin University, Casuarina Campus, Ellengowan Drive, Casuarina, Darwin NT 0909, Australia. [10]Melbourne School of Population and Global Health, The University of Melbourne, PO Box 5266, Burnley VIC 3121, Australia. [11]Melbourne Medical School, The University of Melbourne, Medical Building, Melbourne VIC 3010, Australia.

Received: 29 July 2013 Accepted: 26 September 2013
Published: 17 October 2013

References

1. American Diabetes Associations: **Diagnosis and classification of diabetes mellitus.** *Diabetes Care* 2006, **29:**S43–S48.
2. International Association of Diabetes and Pregnancy Study Groups Consensus Panel: **International Association of Diabetes and Pregnancy Study Groups recommendations on the diagnosis and classification of hyperglycemia in pregnancy.** *Diabetes Care* 2010, **33:**676–682.
3. Hoffman L, Nolan C, Wilson JD, Oats JJN, Simmons D: **Gestational diabetes mellitus—management guidelines.** *The Australasian Diabetes in Pregnancy Society. Med JAust* 1998, **169:**93–97.
4. O'Sullivan JB: **Diabetes mellitus after GDM.** *Diabetes* 1991, **40:**131–135.
5. Australian Institute of Health and Welfare (AIHW): *Templeton M, Pieris-Caldwell I, Gestational Diabetes Mellitus in Australia.* Canberra: AIHW: 2005–06, Diabetes series no. 10 Cat no. CVD 44; 2008.
6. Lindstrom J, Ilanne-Parikka P, Peltonen M, Aunola S, Eriksson JG, Hemio K, Hamalainen H, Harkonen P, Keinanen-Kiukaanniemi S, Laakso M, *et al*: **Sustained reduction in the incidence of type 2 diabetes by lifestyle intervention: follow-up of the Finnish Diabetes Prevention Study.** *Lancet* 2006, **368:**1673–1679.
7. Ratner RE, Christophi CA, Metzger BE, Dabelea D, Bennett PH, Pi-Sunyer X, Fowler S, Kahn SE: **Diabetes Prevention Program Research Group: prevention of diabetes in women with a history of gestational diabetes: effects of metformin and lifestyle interventions.** *J Clin Endocrinol Metab* 2008, **93:**4774–4779.
8. Lee AJ, Hiscock RJ, Wein P, Walker SP, Permezel M: **Gestational diabetes mellitus: clinical predictors and long-term risk of developing type 2 diabetes - A retrospective cohort study using survival analysis.** *Diabetes Care* 2007, **30:**878–883.
9. MacNeill S, Dodds L, Hamilton DC, Armson BA, VandenHof M: **Rates and risk factors for recurrence of gestational diabetes.** *Diabetes Care* 2001, **24:**659–662.
10. Metzger BE: **Long-term outcomes in mothers diagnosed with gestational diabetes mellitus and their offspring.** *Clin Obstet Gynecol* 2007, **50:**972–979.
11. Australian Institute of Health and Welfare (AIHW): *Diabetes Prevalence in Australia.* Canberra: AIHW: Detailed Estimates for 2007–08. Diabetes series no. 17, Volume CVD 56; 2011.
12. Dunstan DW, Zimmet PZ, Welborn TA, de Courten MP, Cameron AJ, Sicree RA, Dwyer T, Colagiuri S, Jolley D, Knuiman M, *et al*: **The rising prevalence of diabetes and impaired glucose tolerance. The Australian diabetes, obesity and lifestyle study.** *Diabetes Care* 2002, **25:**829–834.
13. Begg SJ, Vos T, Barker B, Stanley L, Lopez AD: **Burden of disease and injury in Australia in the new millennium: measuring health loss from diseases, injuries and risk factors.** *MedJ Aust* 2008, **188:**36–40.
14. Vos T, Goss J, Begg S, Nann N: *Projection of Health Care Expenditure by Disease: a Case Study From Australia.* Brisbane: The University of Queensland; 2007.
15. Gillies CL, Abrams KR, Lambert PC, Cooper NJ, Sutton AJ, Hsu RT, Khunti K: **Pharmacological and lifestyle interventions to prevent or delay type 2 diabetes in people with impaired glucose tolerance: systematic review and meta-analysis.** *Br Med J* 2007, **334:**299–302B.
16. Knowler WC, Barrett-Connor E, Fowler SE, Hamman RF, Lachin JM, Walker EA, Nathan DM: **Diabetes Prevention Program Research Group: reduction in the incidence of type 2 diabetes with lifestyle intervention or metformin.** *New Engl J Med* 2002, **346:**393–403.
17. Tuomilehto J, Lindstrom J, Eriksson JG, Valle TT, Hamalainen H, Ilanne-Parikka P, Keinanen-Kiukaanniemi S, Laakso M, Louheranta A, Rastas M, *et al*: **Prevention of type 2 diabetes mellitus by changes in lifestyle among subjects with impaired glucose tolerance.** *New Engl J Med* 2001, **344:**1343–1350.
18. Gerstein HC, Yusuf S, Holman RR, Bosch J, Anand S, Avezum A, Budaj A, Chiasson J, Conget I, Dagenais G, *et al*: **Effect of rosiglitazone on the frequency of diabetes in patients with impaired glucose tolerance or impaired fasting glucose: a randomised controlled trial.** *Lancet* 2006, **368:**1096–1105.
19. Eriksson J, Lindstrom J, Valle T, Aunola S, Hamalainen H, Ilanne-Parikka P, Keinanen-Kiukaanniemi S, Laakso M, Lauhkonen M, Lehto P, *et al*: **Prevention of Type II diabetes in subjects with impaired glucose tolerance: the Diabetes Prevention Study (DPS) in Finland. Study design and 1-year interim report on the feasibility of the lifestyle intervention programme.** *Diabetologia* 1999, **42:**793–801.
20. National Diabetes Service Scheme: [http://www.ndss.com.au/]
21. Bandura A: *Social Learning Theory.* New York: General Learning Press; 1977.
22. Bandura A: *Social Foundation of Thought and Action: A social cognitive theory.* Englewood Cliffs, NJ: Prentice-Hall; 1986.
23. Bandura A: *Self-efficacy: The Exercise of Control.* New York: Worth Publishers; 1997.
24. Schwarzer R: *Self-efficacy in the adoption and maintenance of health behaviors: theoretical approaches and a new model. In Self-efficacy: Thought, Control and Action.* Washington DC: Hemisphere; 1992.
25. Schwarzer R, Fuchs R: *Self-efficacy and health behaviours. Predicting Health Behaviour: Research and Practice with Social Cognition Models.* Buckingham, UK: Open University Press; 1996.
26. Life! Program: http://www.diabeteslife.org.au/.
27. Tolonen H, Kuulasmaa K, Laatikainen T, Wolf H: *European Health Risk Monitoring Project.* Helsinki, Finland: National Public Health Institute (KTL): Recommendation for Indicators, International Collaboration, Protocol and Manual of Operations for Chronic Disease Risk Factor Surveys; 2002. http://www.ktl.fi/publications/ehrm/product2/product2.pdf.
28. Hodge A, Patterson AJ, Brown WJ, Ireland P, Giles G: **The Anti Cancer Council of Victoria FFQ: relative validity of nutrient intakes compared with weighed food records in young to middle-aged women in a study of iron supplementation.** *Aust N Z J Public Health* 2000, **24:**576–583.
29. Australian Institute of Health and Welfare (AIHW): *The Active Australia Survey: a Guide and Manual for Implementation.* Canberra: AIHW: Analysis and Reporting; 2003.

30. Levesque CS, Williams GC, Elliot D, Pickering MA, Bodenhamer B, Finley PJ: **Validating the theoretical structure of the Treatment Self-Regulation Questionnaire (TSRQ) across three different health behaviors.** *Health Educ Res* 2007, **22**:691–702.
31. Renner B, Schwarzer R: **Risk and health behaviors: documentation of the Scales of the Research Project "Risk Appraisal Consequences in Korea" (RACK).** In *Risk and health behaviors: documentation of the Scales of the Research Project "Risk Appraisal Consequences in Korea" (RACK).* International University Bremen & Freie Universität Berlin: 2nd edition; 2005:1–55.
32. Schwarzer R, Renner B: *Health-specific self-efficacy scales*; 2008:1–21. http://userpage.fu-berlin.de/~health/healself.pdf.
33. Zimet G, Dahlem N, Zimet S, Farley G: **The Multidimensional Scale of Perceived Social Support.** *J Pers Assess* 1988, **52**:30–41.
34. Richardson J, Sinha K, Iezzi A, Khan M: *Modelling the Utility of Health States with the Assessment of Quality of Life (AQoL) 8D Instrument: Overview and utility scoring algorithm.* Melbourne: Monash University Centre for Health Economics; 2011.
35. Kroenke K, Spitzer RL, Williams JBW: **The PHQ-9 - Validity of a brief depression severity measure.** *J Gen Intern Med* 2001, **16**:606–613.
36. Laatikainen T, Dunbar JA, Chapman A, Kilkkinen A, Vartiainen E, Heistaro S, Philpot B, Absetz P, Bunker S, O'Neil A, *et al*: **Prevention of type 2 diabetes by lifestyle intervention in an Australian primary health care setting: Greater Green Triangle (GGT) Diabetes Prevention Project.** *BMC Public Health* 2007, **7**:249. doi:doi: 210.1186/1471-2458-1187-1249.
37. O'Sullivan J: **The Boston Gestational Diabetes Studies: review and perspectives.** In *Carbohydrate Metabolism in Pregnancy and the Newborn.* London: Springer-Verlag: Sutherland H, Stowers J, Pearson D; 1989:287–294.

doi:10.1186/1745-6215-14-339
Cite this article as: Shih *et al.*: Mothers After Gestational Diabetes in Australia Diabetes Prevention Program (MAGDA-DPP) post-natal intervention: study protocol for a randomized controlled trial. *Trials* 2013 **14**:339.

Submit your next manuscript to BioMed Central and take full advantage of:

- **Convenient online submission**
- **Thorough peer review**
- **No space constraints or color figure charges**
- **Immediate publication on acceptance**
- **Inclusion in PubMed, CAS, Scopus and Google Scholar**
- **Research which is freely available for redistribution**

Submit your manuscript at
www.biomedcentral.com/submit

BioMed Central
