# Supplementary material for: Mothers after Gestational Diabetes in Australia (MAGDA): A Randomised Controlled Trial of a Postnatal Diabetes Prevention Program
Source: PLoS Med. 2016 Jul 26;13(7):e1002092. doi: 10.1371/journal.pmed.1002092 (PMC4961439; doi:10.1371/journal.pmed.1002092)
Supplement: S3 Text — (PDF) [file pmed.1002092.s003.pdf]

Shih et al. Trials 2014, 15:259
http://www.trialsjournal.com/content/15/1/259

TRIALS

UPDATE Open Access

Mothers After Gestational Diabetes in Australia Diabetes Prevention Program (MAGDA-DPP) post-natal intervention: an update to the study protocol for a randomized controlled trial

Sophy TF Shih[1], Nathalie Davis-Lameloise[2], Edward D Janus[2,3], Carol Wildey[2,4], Vincent L Versace[2], Virginia Hagger[5], Dino Asproloupos[2,4], Sharleen L O'Reilly[6], Paddy A Phillips[7,8], Michael Ackland[9], Timothy Skinner[10], Jeremy Oats[11], Rob Carter[1], James D Best[12], James A Dunbar[2*] and On behalf of the MAGDA Research Group

Abstract

Background: The Mothers After Gestational Diabetes in Australia Diabetes Prevention Program (MAGDA-DPP) is a randomized controlled trial (RCT) that aims to assess the effectiveness of a structured diabetes prevention intervention for women who had gestational diabetes.

Methods/Design: The original protocol was published in *Trials* (http://www.trialsjournal.com/content/14/1/339). This update reports on an additional exclusion criterion and change in first eligibility screening to provide greater clarity. The new exclusion criterion "surgical or medical intervention to treat obesity" has been added to the original protocol. The risks of developing diabetes will be affected by any medical or surgical intervention as its impact on obesity will alter the outcomes being assessed by MAGDA-DPP. The screening procedures have also been updated to reflect the current recruitment operation. The first eligibility screening is now taking place either during or after pregnancy, depending on recruitment strategy.

Trial registration: Australian New Zealand Clinical Trials Registry ANZCTRN 12610000338066.

Keywords: Gestational diabetes, Lifestyle intervention, Post-natal, Type 2 diabetes prevention

Update

Introduction

The Mothers After Gestational Diabetes in Australia Diabetes Prevention Program (MAGDA-DPP) is a randomized controlled trial (RCT) aiming to assess the effectiveness of a structured diabetes prevention intervention for women with previous gestational diabetes (GDM). The MAGDA-DPP trial offers an evidence-based structured lifestyle modification group intervention for such women. The objectives of MAGDA-DPP are that the intervention will result in favourable changes, relative to usual care, in clinical-, behavioural-, and patient-relevant outcomes. In addition, MAGDA-DPP aims to identify individual characteristics predictive of successful outcomes.

The MAGDA-DPP intervention is coordinated from Deakin University (Melbourne Campus). The project includes multiple study partners, consisting of two State Governments, three universities, and two non-government organizations. Ethical approvals have been obtained from multiple ethics authorities of the MAGDA project partners for the original study, with the lead ethics review authority being Deakin University Human Research Ethics Committee (reference number 2010–005).

The original protocol for the study has been presented by Shih et al. [1]. Following the publication of the protocol, amendments have been made to reflect the changes to initial screening and recruitment processes and provide greater protocol clarity. The two changes are outlined below. The amendments of the protocol have also

* Correspondence: director@greaterhealth.org
[2]Greater Green Triangle University Department of Rural Health, Flinders University, PO Box 423, 3280 Warrnambool, VIC, Australia
Full list of author information is available at the end of the article

© 2014 Shih et al.; licensee BioMed Central Ltd. This is an Open Access article distributed under the terms of the Creative Commons Attribution License (http://creativecommons.org/licenses/by/2.0), which permits unrestricted use, distribution, and reproduction in any medium, provided the original work is properly credited. The Creative Commons Public Domain Dedication waiver (http://creativecommons.org/publicdomain/zero/1.0/) applies to the data made available in this article, unless otherwise stated.

been approved by Deakin University Human Research Ethics Committee in 2014. Informed consents have been obtained from all study participants.

Selection of participants

A new exclusion criterion has been added into the protocol "surgical or medical intervention to treat obesity". Any medical or surgical intervention that impacts on the level of obesity, such as gastric banding, will alter the risks of developing diabetes and thus impact the outcomes being assessed by the MAGDA-DPP trial. Consequently, potential participants undergoing such an intervention will need to be excluded. The exclusion criteria are now: (i) pre-existing diabetes (Type 1 diabetes mellitus or T2DM); (ii) cancer (not in remission); (iii) severe mental illness; (iv) substance abuse (illicit drugs); (v) myocardial infarction in the last three months; (vi) difficulty with English; (vii) involvement in a post-natal lifestyle-based intervention or a trial which may impact primary clinical outcomes; (viii) pregnancy at post-natal baseline testing or at any point during the 12-months of study involvement; and (ix) surgical or medical intervention to treat obesity.

Additionally, the screening procedures have been updated to reflect current practice. As a result of slow recruitment initially, mail-outs were sent to women with recent GDM history in selected geographic areas. Following the mail-out, a large proportion of participants were recruited and their first eligibility screening was conducted over the phone at various post-partum time points. Therefore, the first eligibility screening took place either during or after pregnancy depending on recruitment strategy. This represents a change to the original protocol which stated "*Once women diagnosed with GDM express interest in the study, the recruiter (MAGDA-DPP project manager or research assistant) will conduct the first eligibility screening generally prior to women delivering their babies*".

Competing interests

The authors declare that they have no competing interests.

Authors' contributions

SS: Study design, manuscript writing, revision, and final approval of the version to be published; NDL: Study design, manuscript writing, revision, and final approval of the manuscript; EJ: Study design, obtaining funding for the study, revision, and final approval of the manuscript; CW: Amendment of the study protocol, manuscript writing, revision, and final approval of the manuscript; VV: Study design, revision, and final approval of the manuscript; VH: Study design, revision, and final approval of the manuscript; DA: Amendment of the study protocol, revision, and final approval of the manuscript; SO: Study design, revision, and final approval of the manuscript; PP: Study design, obtaining funding for the study, revision, and final approval of the manuscript; MA: Study design, obtaining funding for the study, revision, and final approval of the manuscript; TS: Study design, revision and final approval of the manuscript; JO: Study design, obtaining funding for the study, revision, and final approval of the manuscript; RC: Study design, obtaining funding for the study, revision, and final approval of the manuscript; JB: Study design, obtaining funding for the study, revision, and final approval of the manuscript; and JD: Study design, obtaining funding for the study, general supervisor of the study, revision, and final approval of the manuscript.

Acknowledgements

The study was funded by a Partnerships for Better Health Grant from the National Health and Medical Research Council (grant number: 533956) with financial and in-kind contributions from Victoria State Government, South Australia State Government, and Diabetes Australia Victoria. We would like to acknowledge Dr Rosalind Lau and Professor Prasuna Reddy for the initial development of the study protocol, as well as Kate Schlicht, Associate Professor John Reynolds, and Professor Catherine Bennett for their advice on aspects of psychology, statistics and epidemiology respectively. We would also like to acknowledge the contributions of the MAGDA Manual Training Committee and MAGDA RCT Working Group to the intervention handbooks and the study protocol revision. The views expressed in this manuscript are those of the authors and do not necessarily represent the views of, nor should be attributed to, the National Health and Medical Research Council, Department of Health Victoria, South Australia Health, and Diabetes Australia Victoria.

Author details

[1]Deakin Health Economics, Population Health Strategic Research Centre, Faculty of Health, Deakin University, 221 Burwood Highway, 3125 Burwood, VIC, Australia. [2]Greater Green Triangle University Department of Rural Health, Flinders University, PO Box 423, 3280 Warrnambool, VIC, Australia. [3]Department of Medicine, North West Academic Centre, The University of Melbourne, Western Centre for Health Research and Education, Western Health, 176 Furlong Rd, 3021 St Albans, VIC, Australia. [4]Deakin Health Services Implementation Research Unit, Population Health Strategic Research Centre, Faculty of Health, Deakin University, 221 Burwood Highway, 3125 Burwood, VIC, Australia. [5]Diabetes Australia – Victoria, Melbourne, 570 Elizabeth Street, 3000 Melbourne, VIC, Australia. [6]Centre for Physical Activity and Nutrition Research, Faculty of Health, Deakin University, 221 Burwood Highway, 3125 Burwood, VIC, Australia. [7]South Australia Health, GPO Box 2100, 5001 Adelaide, SA, Australia. [8]Department of Medicine, Flinders University, GPO Box 2100, 5001 Adelaide, SA, Australia. [9]Office of the Chief Health Officer, Department of Health, 14/50 Lonsdale Street, 3000 Melbourne, VIC, Australia. [10]Psychological and Clinical Sciences, Charles Darwin University, Casuarina Campus, Ellengowan Drive, Casuarina, 0909 Darwin, NT, Australia. [11]Melbourne School of Population and Global Health, The University of Melbourne, PO Box 5266, 3121 Burnley, VIC, Australia. [12]Melbourne Medical School, Level 2 West Wing, Medical Building (181), 3010 The University of Melbourne, Melbourne, VIC, Australia.

Received: 3 February 2014 Accepted: 13 June 2014
Published: 30 June 2014

Reference

1. Shih STF, Davis-Lameloise N, Janus ED, Wildey C, Versace VL, Hagger V, Asproloupos D, O'Reilly S, Phillips PA, Ackland M, Skinner T, Oats J, Carter R, Best JD, Dunbar JA, MAGDA Research Group: **Mothers After Gestational Diabetes in Australia Diabetes Prevention Program (MAGDA-DPP) post-natal intervention: study protocol for a randomized controlled trial.** *Trials* 2013, **14**:339.

doi:10.1186/1745-6215-15-259
Cite this article as: Shih *et al.*: **Mothers After Gestational Diabetes in Australia Diabetes Prevention Program (MAGDA-DPP) post-natal intervention: an update to the study protocol for a randomized controlled trial.** *Trials* 2014 **15**:259.
