# Supplementary material for: Mothers after Gestational Diabetes in Australia (MAGDA): A Randomised Controlled Trial of a Postnatal Diabetes Prevention Program
Source: PLoS Med. 2016 Jul 26;13(7):e1002092. doi: 10.1371/journal.pmed.1002092 (PMC4961439; doi:10.1371/journal.pmed.1002092)
Supplement: S4 Text — (PDF) [file pmed.1002092.s004.pdf]

# Mothers After Gestational Diabetes in Australia (MAGDA Study)

## Statistical Analysis Plan
**Version 1.0**

### Scope

This Statistical Analysis Plan (SAP) has been prepared to define and guide the statistical analyses that will be included in a First Interpretable Results (FIR) report for the Study Management Committee. The report will be restricted to the primary and secondary outcomes and the five goals of the Life! Program as described in the protocol paper (Shih et al, 2013). The analyses for the FIR will be conducted on a locked (i.e. read-only) version of the study database and the Trial Statistician will be unblinded to participants' treatment allocations when this version of the database is made available. The FIR results for the primary outcomes will also be used in the planned economic evaluation of the MAGDA Study in the trial-based cost-effectiveness analysis as well as to model health outcomes (life-years and QALYs) over remaining life expectancy. The methodological approach proposed for the economic evaluation is described in a separate document but it will rely upon intention to treat (ITT) statistical analyses of both the demographic data and the primary clinical parameters (refer Section 2.4) with a sensitivity analysis restricted to subjects in the per-protocol set (PPS).

Additional exploratory analyses will be conducted and reported in the Final Report. The SAP will be revised and approved prior to commencing these other analyses required for the Final Report. Analyses for the Final Report will also be conducted on a locked (i.e. read-only) version of the study database. Analyses reported in the FIR will not be revised for the Final Report unless extraordinary circumstances (documented in a Database Lock Report) require this.

### SAP History

| Version No. | Date | Authors | Reason |
|---|---|---|---|
| 1 | 21st of September 2015 | Dr Vin Versace and Dr John Reynolds | SAP for analyses for the FIR report |
| 2 | | | |

### SAP Approval

| Version No. | Date Approved | Approver | Signature of Approver |
|---|---|---|---|
| 1 | | Prof James Dunbar | 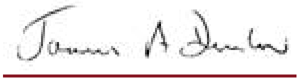 |
| 2 | | | |

DRAFT

# 1. Introduction

The Mothers After Gestational Diabetes in Australia (MAGDA) aims to develop and implement a macrolevel system change to reduce the risk of progression to T2DM for women with previous GDM. The project consists of four components: (1) evaluation of the National Gestational Diabetes Register (NGDR) through data linking Victorian and South Australia perinatal databases with the Register and pathology results; (2) an intervention to reduce progression to T2DM; (3) a health economics evaluation of the register and intervention; and (4) an understanding of how to implement the register and follow-up in general practice.

### 1.1 MAGDA Study 2 – randomised controlled trial RCT

The study protocol for the MAGDA Study 2 randomised controlled trial has been detailed elsewhere by Shih et al. (2013). Briefly, the MAGDA-DPP is a prospective, open RCT to assess the effectiveness of a structured diabetes prevention program (DPP) for women who have had GDM. This trial has an intervention group participating in a DPP and a control group receiving usual care from their general practitioners (GPs) during the same time period. Women will be recruited from two Australian State (Shih et al. (2013).

This document provides details of the proposed Statistical Analysis Plan for the first interpretable results (FIR) for MAGDA Study 2 – the randomised controlled trial. It will not cover the other three components of the overall study described in the Introduction. Specifically it covers the primary endpoints (changes in FPG, weight, and waist), secondary endpoints (2-hour OGTT, triglycerides, HDL, LDL, blood pressure, depression, physical activity and diet). Physical activity and diet will be assessed using the goals of the Life! program that were developed based upon the experiences reported by two clinical trials of prevention of T2DM with lifestyle modification (described by Davis-Lameloise et al, 2013). The goals to be assessed are: (1) no more than 30% energy from fat; (2) no more than 10% energy from saturated fat; (3) at least 15 g/1,000 kcal fibre intake; (4) at least 30 minutes/day moderate intensity physical activity; and (5) at least 5% reduction in body weight.

## 1.2 Study Objectives

The working hypotheses of the MAGDA RCT are that the intervention results in statistically and clinically significant changes (relative to usual care) in clinical, behavioural, and patient relevant outcomes,

## 1.3 Design

### Inclusion criteria

The inclusion criterion for women is diagnosis of GDM in their most recent pregnancy, with GDM defined by the Australasian Diabetes in Pregnancy Society (ADIPS) criteria: fasting plasma glucose (FPG) of 5.5 mmol/L or higher, and/or 2 hour glucose of 8.0 mmol/L or higher on a 75 g oral glucose tolerance test (OGTT) [3], or a glucose challenge test (GCT) result of 12.0 mmol/L or higher.

### Exclusion criteria

Individuals meeting any of the exclusion criteria outlined below at baseline will be excluded from the study.
Exclusion criteria:

(1) established diabetes (Type 1 or 2 diabetes mellitus);
(2) cancer (not in remission);
(3) severe mental illness in the last three months;
(4) substance abuse (illicit drugs) in the last three months;

(5) myocardial infarction in the preceding three months;
(6) difficulty with English;
(7) involvement in another post-natal intervention trial or a similar clinical trial; and
(8) pregnancy at post-natal baseline testing or at any point during the 12months of study involvement.
(9) surgical or medical intervention to treat obesity

## 1.4 Intervention

This is a parallel group study, with the intervention group receiving a diabetes prevention program for 12 months and the control group receiving usual care from their General Practitioners (GPs) during the same time period. All women recruited into the study will be followed up for 12 months. At baseline, the women will undergo an assessment consisting of blood tests, anthropometric measurements, and self-reported assessment of health status, diet, physical activity, quality of life, depression and risk perception. The intervention group will be assessed at baseline, at three and 12 months. The control group will be assessed at baseline and 12 months (Shih et al, 2013).

The MAGDA-DPP intervention is coordinated by Deakin University (Melbourne Campus) and is supported by a ‘Partnerships for Better Health’ Grant from the National Health and Medical Research Council of Australia (NHMRC). The project includes multiple study partners, consisting of two state governments, three universities and two non-government organizations.

DRAFT

# 2. General Considerations

## 2.1 Time windows

The date of randomization is considered to be the first day on the study (Study Day 1). Randomization only occurs after final eligibility has been established. The “T1” assessment is also termed the “baseline” assessment. The references to intervention activities below should be read in conjunction with Figure 2 from Shih et al. (2013).

**T1 window**

For subjects randomized to the “usual care” arm of the study, the “T1” assessment is the last assessment of any variable (e.g. weight in kg) in the window that extends from the date of randomization to the 28 days prior to randomization (inclusive). For subjects randomized to the “intervention” arm, the “T1” assessment is also the last assessment in this window but with the added proviso that the assessment occurred <u>before</u> the “initial individual session”.

**T2 window**

For subjects randomized to the intervention arm, the T2 (or 3-month) assessment is the assessment nearest to Study Day 91 in the interval from session “G5” (on, or after, either the actual date of G5 or the scheduled date if G5 was skipped) to 60 days after session “G5” provided the assessment was before session “P1” (either the actual date of P1 or the scheduled date if P1 was skipped). For subjects in the intervention arm without a T2 assessment that meets the definition of the T2 window, the post-baseline assessment closest to Study Day 91 but before Study Day 270 will be used in the intention to treat analysis; if no assessment meets this relaxed criterion, the procedure for handling missing values will apply (see Section 3.1).

**T3 window**

For subjects randomized to the “usual care” arm of the study, the “T3” assessment is the last assessment of any variable in the window that extends from Study Day 270 (approximately 9months on study) to Study Day 548 (approximately 18 months on study) inclusive. For subjects randomized to the “intervention” arm, the “T3” assessment is also the last assessment in this window but with the added proviso that the assessment occurred after session “P2” (either the actual date of P2 or the scheduled date if P2 was skipped). For subjects without a T3 assessment that meets this definition of the T3 window, the last assessment on, or after, Study Day 270 will be used in the intention to treat analysis; if no assessment meets this relaxed criterion, the procedure for handling missing values will apply (see Section 3.1).

## 2.2 Overview of data collection for the intervention group

**Table 1.** Summary of data collected at each time point during the trial.

| | Unit/Scale | Baseline (BL) | 3 months (T2) | 12 months (T3) |
|---|---|---|---|---|
| **Clinical Test Form** | | | | |
| Weight | Kg | ✓ | ✓ | ✓ |
| Waist circumference | Cm | ✓ | ✓ | ✓ |
| Hip circumference | Cm | ✓ | | ✓ |
| Blood pressure | mmHg | ✓ | | ✓ |
| Blood pressure medication | Binary (Y/N) | Binary (Y/N) | Binary (Y/N) | Binary (Y/N) |
| 2 hour glucose | mmol/L | ✓ | | ✓ |
| **Health Status Questionnaire** | Demographic information | ✓ | | |
| **Pathology** | | | | |
| FPG | mmol/L | ✓ | ✓ | ✓ |
| HbA1C | mmol/L | ✓ | ✓ | ✓ |
| 2 hour glucose | mmol/L | ✓ | | ✓ |
| Total Cholesterol | mmol/L | ✓ | ✓ | ✓ |
| Triglycerides | mmol/L | ✓ | ✓ | ✓ |
| HDL | mmol/L | ✓ | ✓ | ✓ |
| LDL | mmol/L | ✓ | ✓ | ✓ |
| Estimated Average Glucose (eAG) | mmol/L | | ✓ | |
| | | **Baseline Questionnaire** | **3 month Questionnaire** | **12 month Questionnaire** |
| Demographics | | ✓A | | ✓A |
| Health Status | | ✓B | | ✓B |
| Risk Perception | Ordinal (1-10) | ✓C | | ✓C |
| Physical Activity: Part 1 | Ordinal (1-4) | ✓E | | ✓E |
| Physical Activity: Part 2 | Ordinal (1-4) | ✓E | | ✓E |
| Physical Activity: Part 3 | Ordinal (1-7) | ✓E | | ✓E |
| Physical Activity: (The Active Australia) | Frequency, time data, and Ordinal 1-5 | ✓F | | ✓F |
| Physical Activity: (Overall) | Ordinal (1-8) | ✓F | | ✓F |
| Eating Habits: Part 1 | Ordinal (1-4) | ✓D | | ✓D |
| Eating Habits: Part 2 | Ordinal (1-4) | ✓D | | ✓D |
| Eating Habits: Part 3 | Binary (Y/N) or Ordinal (1-6) | ✓D (Y/N) | | ✓D (Y/N) |
| Eating Habits: Part 4 | Ordinal (1-7) | ✓D | | ✓D |
| Social Support | Ordinal (1-7) | ✓I | | ✓I |
| Quality of Life | Ordinal (1-4;1-5;1-6) | ✓G | | ✓G |
| Psychological Wellbeing | Ordinal (0-3) | ✓H | | ✓H |
| Food Frequency Questionnaire | | ✓ | | ✓ |
| Use of Health Care | Short answer | | | ✓ |
| Program Feedback – | Qualitative | | ✓ | ✓ |
| Barriers and Enablers | Qualitative | | | |

✓ Data collected at point in project timeline
A;B;C;D;E;F;G;H;I corresponds to respective section of questionnaire at each time point

DRAFT

## 2.3 Analysis sets

### Full Analysis Set (FAS)

The Full Analysis Set (FAS) is defined according to the Intention to Treat (ITT) principle. The FAS consists of all randomized subjects analysed according to the study arm to which they were assigned at randomization, with the possible exception of subjects who were randomized to the study in error.

### Per-Protocol Set (PPS)

The Per-Protocol Set (PPS) consists of all patients in the FAS without a major protocol deviation.  Major protocol deviations include, but are not limited to:

- Subject was assigned to the intervention arm but did not attend any group sessions (i.e. the minimal exposure requirement is attendance at the individual session and one group session)
- Subject did not have a post-baseline assessment that meets the time-window definitions.
- Subject became pregnant while on the study.
- An eligibility criterion that was deemed to have been met at the time of randomization was later found not to have been met

All protocol deviations will be finalized before database lock, and an indicator variable created in the derived dataset by the unblinded Data Manager. Reasons for excluding subjects from the PPS will be tabulated by study arm in both the FIR and the Final Report.

The PPS is used for a sensitivity analysis of the primary endpoints.

## 2.4 Primary Endpoints

The primary endpoints will be weight (kg), waist circumference (cm), and fasting plasma glucose (mmol/L).

DRAFT

# 3. Statistical Methods

## 3.1 Statistical Analyses of Primary Endpoints

The statistical analyses of the primary endpoints will be conducted on the FAS.

Repeated measures analysis of variance (ANOVA), or a mixed model analysis, will be used to analyse each primary endpoint. The null hypothesis, that there is no interaction between assessment time (T1 and T3) and group (Control and Intervention) will be tested using an F-test conducted at the 5% significance level ($\alpha=0.05$). The p-value for this test will be reported together with a two-way table of means (time by group) and their associated 95% confidence intervals. Comparisons of the assessment times (the T3 versus T1 differences) within each treatment group (Control or Intervention) will be based on t-tests that utilise the standard errors of the differences that are computed as part of the repeated measures analysis. The p-values resulting from these t-tests will be reported in the FIR report regardless of the outcome of the global F-test for the interaction. Diagnostic plots of residuals will be checked for each analysis of a primary endpoint to assess whether or not there are departures from the assumptions required for use of the F and t-tests. Where assumptions have been violated (such as the assumption of homogeneity of the residual variance) an appropriate transformation will be selected (such as the log transformation) and the analysis will be rerun on the transformed data; the p-values for the F and t-tests from the analysis on the transformed scale will be reported together with a two-way table of the back-transformed means and their associated back-transformed 95% confidence intervals.

### 3.1.1 Missing values in the primary endpoints

In the event that some subjects have missing assessments at T3, the analysis of variance will be replaced by a mixed model analysis using the method of residual (or restricted) maximum likelihood (REML). F-tests, t-tests, diagnostic checks of assumptions and possible re-analyses of transformed endpoints will proceed as outlined above, the only difference being that the reported means are no longer equivalent to simple arithmetic means but are predicted means that are recovered from the mixed model that has been fitted to the data. These analyses will be conducted on the FAS.

### 3.1.2 Per-protocol analyses

In the event that the FAS differs from the PPS, repeated measures ANOVAs will also be conducted on the PPS for each primary endpoint. The PPS excludes subjects who do not have a post-baseline assessment (a form of case-wise deletion) and so ANOVA can be used for analyses of the PPS. If inferences from the analyses of the PPS differ markedly from the inferences from the analyses of the FAS, this will be regarded as an indication that compliance with the protocol has an impact on claims about the effect of the intervention, nevertheless the analysis of the FAS will be regarded as the primary analysis.

### 3.1.3 Subgroup analyses of the primary endpoints (Influence of recruitment method)

During the accrual phase of the trial two distinct methods of recruitment were used: (1) recruitment through participating hospitals (antenatal), referrals from private healthcare providers (antenatal and postnatal) and retrospective database mining of hospital records (postnatal), and (2) mailed leaflets from the national Diabetes Services Scheme (NDSS), using data from the National Gestational Diabetes Register (NGDR) to relevant postcodes in Adelaide and Melbourne (antenatal and postnatal). It is anticipated that the randomisation will ensure that no major confounding of the type of subject in each treatment arm will occur. It is acknowledged that the two sub-populations might respond differently to the intervention

as those receiving the mail out may be, on average, a longer period postpartum than subjects recruited through other avenues.

To assess the influence of recruitment method on primary and secondary endpoints the fixed effects part of the mixed linear model will be extended to include treatment (intervention or control) crossed with register (yes or no) crossed with time (T1 or T3) (i.e. Treatment + Register + Time +Treatment*Register + Treatment*Time = Treatment*Register*Time).

Regardless of the significance of the interaction terms, the Treatment*Time interaction contrast of the four means in each sub-population stratum (i.e. Register, yes or no) will be tested for a significant difference from zero. These two t-tests will have a conservative Bonferroni adjustment made and will be carried out at the 2.5% significance level ($\alpha$=0.025) because they are conducted without the protection of a significant global F-test of the three-way interaction.

ANOVA will be used if there are no missing assessments, otherwise REML will be used.

### 3.1.4 Tracking intervention participants (primary endpoints)

Repeated measures analysis of variance (ANOVA), or a mixed model analysis (REML), will be used to analyse differences between assessment time periods (T1, T2 and T3) for each primary endpoint. The null hypothesis, that there is no difference between assessment time periods will be tested using a global F-test conducted at the 5% significance level ($\alpha$=0.05). Post hoc testing, via t-tests that utilise the standard errors of differences estimated in the global analysis, will be carried out at the 5% significance level to identify where pairwise differences exist between times.

## 3.2 Statistical Analyses of Secondary Endpoints

Analyses of the secondary endpoints 2-hour OGTT, triglycerides, HDL, LDL, systolic and diastolic blood pressure, and depression will follow the methods described in section 3.1 Statistical Analyses of Primary Endpoints.

Blood pressure measurements may be taken up to three times at a visit.  The following rule will be used to create a single, summary, measurement (for each of systolic and diastolic blood pressure) at each visit: Two measurements are taken one minute apart and the mean is used for data analysis. If the second measurement differs by more than 10 mmHg systolic or 6 mmHg diastolic, a third measurement is taken one minute later. The mean of the closest two systolic and diastolic measurements is used for analysis (Davis-Lameloise et al, 2013).

### 3.2.1 Five goals of the Life! Program

The secondary endpoints of diet and physical activity are covered by the goals of the Life! program.

Analyses of the five goals of the Life! Program will be carried out on variables derived from anthropometric measures (at least 5% weight loss), the physical activity assessment (at least 30 minutes/day moderate intensity physical activity) and the Food Frequency Questionnaire (no more than 30% energy from fat; no more than 10% energy from saturated fat; at least 15 g/1,000 kcal fibre intake). Assessing participants against the goals will be based on derived variables from the Dietary Questionnaire for Epidemiological Studies Version 2 (DQES v2) User Information Guide (Cancer Council, 2014) (Appendix – Derived variables).



DRAFT

A summary of the proportion of participants achieving each of the goals at T1 and T3 will be provided. Each goal will be analysed individually as a binary outcome (i.e. the goal was or was not achieved). Generalised Estimating Equations (GEE) with a logit link function will be used to assess goals 1, 2, 3 and 4 (See Table 2). Goal 5 will be assessed using a two-sample binomial test to compare the proportions in the intervention and control groups who achieve the goal at 12 months. The combined score of the number of goals achieved at 12 months will be assessed using an ANOVA. This score ranges from 0 to 5. Analyses of each goal and the analysis of the combined score will be carried out on non-missing values. A sensitivity analysis, in which missing assessments of a goal will be deemed to indicate non-achievement of the goal will be conducted for each goal and also for the combined score.

#### 3.2.1 Tracking intervention Participants (secondary endpoints)

Tracking of intervention participants for the secondary endpoints 2-hour OGTT, triglycerides, HDL, LDL, systolic and diastolic blood pressure, and depression will follow the methods described in section 3.1 Tracking Intervention Participants.

#### 3.1.2 Subgroup analyses of the secondary endpoints (Influence of recruitment method)

Subgroup analyses examining the influence of recruitment method for the secondary endpoints 2-hour OGTT, triglycerides, HDL, LDL, systolic and diastolic blood pressure, and depression will follow the methods described in section 3.1.3 Subgroup analyses of the primary endpoints (Influence of recruitment method).

## 3.3 Demographic and health-related information

The demographic Questionnaire data collected includes healthcare card holder status, country of birth, language(s) spoken at home, cultural heritage, ATSI status, marital status, living arrangements, number of children and their ages, highest level of education achieved, current employment status, occupation, income level, breastfeeding (past and present) and sleeping patterns (amount, quality and disruptions to).

The Health Status Questionnaire data collected includes family history of Type 2 Diabetes, previous pregnancies and Gestational Diabetes diagnoses, diagnoses of depression and other health related reasons restricting participation in physical activity sessions. Data is also collected on smoking status and amount, alcohol consumption and other specific medical conditions and allergies.

Mean, median, minimum, maximum and standard deviations will be generated for the variables age and years of fulltime education. Age will also be presented as frequency data in 5-year age groups. Employment status, smoking status, education level, income level will be presented as proportions. These results will be presented as three columns: (1) control participants; (2) intervention participants; and (3) all participants in the trial. Subgroup analysis will be undertaken to compare gender.

Continuous variables will be assessed using independent t-tests. Where assumptions have been violated, a variance-stabilising transformation will be applied (e.g. Log) and the

DRAFT

analyses rerun. If this is unsuccessful non-parametric methods will be applied. Differences in proportions will be assessed using chi-square tests.

DRAFT

## 4. List of Tables

**Table 1.** Baseline characteristics of control participants, intervention participants, and all participants in the trial

| | Control (n=) | Intervention (n=) | All participants (n=) |
|---|---|---|---|
| Age (years) | | | |
| N | xx | xx | xx |
| Mean (standard deviation) | xx.x (xx.x) | | |
| Median | xx.x | | |
| Age – n (%) | | | |
| <= 24 years | xx (xx.x) | | |
| >25 to <= 29 years | xx (xx.x) | | |
| >30 to <= 34 years | | | |
| >35 to <= 39 years | | | |
| >40 years | | | |
| Waist circumference (cm) | | | |
| N | | | |
| Mean (standard deviation) | | | |
| Median | | | |
| Waist circumference – n (%) | | | |
| <80 | | | |
| 80 and <90 | | | |
| 90 and <100 | | | |
| 100 and <110 | | | |
| 110 and < 120 | | | |
| 120+ | | | |
| BMI | | | |
| N | | | |
| Mean (standard deviation) | | | |
| Median | | | |
| BMI – n (%) | | | |
| <20 (Underweight) | | | |
| 20.00 to < 25 (Normal) | | | |
| 25 to < 30 (Overweight) | | | |
| 30+ (Obese) | | | |
| Weight (kg) | | | |
| N | | | |
| Mean (standard deviation) | | | |
| Median | | | |
| Weight – n (%) | | | |
| <60 | | | |
| 60 to <70 | | | |
| 70 to <80 | | | |
| 80 to <90 | | | |
| 90 to <100 | | | |
| 100+ | | | |
| Education | | | |
| No education | | | |
| Primary | | | |
| Secondary | | | |
| Tertiary | | | |
| Other | | | |
| Income* – n (%) | | | |
| Low[1] | | | |
| Medium[2] | | | |
| High[3] | | | |

DRAFT

| |
|---|
| Current smokers – n (%) |
| Employment – n (%) |
| Full time employed |
| Part time employed |
| Casual employed |
| Unemployed |
| Home duties |
| Other |
| PHQ-9 |
| Minimal depression (0-4) |
| Mild depression (5-9) |
| Moderate depression (10-14) |
| Moderately severe depression (15-19) |
| Severe depression (20-27) |

[1]**Low income range**

- People receiving a full or part pension.
- People with a health care card.
- Single people with a before-tax income of under $34,232.
- Couples with a before-tax income of under $55,692.
- Families with one child with a before-tax income of under $61,225 (plus $5,533 for each extra dependent child).
- Any of the above who face additional costs (due to disability and/or other factors) which reduces their income to this level.

[2]**Medium income range**

- Single people with a before-tax income greater than $34,232 but less than $73,334.
- Couples with a before-tax income greater than $55,692 but less than $98,036.
- Families with one child with a before-tax income greater than $61,225 but less than $103,240 (plus $5,530 for each extra dependent child.)
- Any of the above who face additional costs (due to disability and/or other factors) which reduces their income to this level.

[3]**High income range**

- Single people with a before-tax income of more than $73,334.
- Couples with a before-tax income of more than $98,036.
- Families with one child with a before-tax income of more than $103,240 (plus $5,530 for each extra dependent child).

**Table 2.** Two-way table of predicted means (± 95%CI) of time (Baseline and 12 months) and group (Control and Intervention) – Primary and secondary endpoints.

| | Control | Intervention | Difference (±LSD) |
|---|---|---|---|
| Weight (kg) (P=x.xxx)[1] | | | |
| Baseline | xx.x (xx.x, xx.x) | xx.x (xx.x, xx.x) | xx.x (xx.x) |
| 12 months | xx.x (xx.x, xx.x) | xx.x (xx.x, xx.x) | xx.x (xx.x) |
| Difference (±LSD) | xx.x (xx.x) | xx.x (xx.x) | |
| Waist circumference | | | |
| Baseline | | | |
| 12 months | | | |
| Difference (±LSD) | | | |
| FPG (mmol/L) (P=x.xxx)[1] | | | |
| Baseline | | | |

DRAFT

| | | | |
|---|---|---|---|
| 12 months | | | |
| Difference (±LSD) | | | |
| 2 hour glucose (mmol/L) (P=x.xxx)[1] | | | |
| Baseline | | | |
| 12 months | | | |
| Difference (±LSD) | | | |
| Systolic BP (mmHg) (P=x.xxx)[1] | | | |
| Baseline | | | |
| 12 months | | | |
| Difference (±LSD) | | | |
| Diastolic BP (mmHg) (P=x.xxx)[1] | | | |
| Baseline | | | |
| 12 months | | | |
| Difference (+LSD) | | | |
| Total cholesterol (mmol/L) (P=x.xxx)[1] | | | |
| Baseline | | | |
| 12 months | | | |
| Difference (±LSD) | | | |
| Triglycerides (mmol/L) (P=x.xxx)[1] | | | |
| Baseline | | | |
| 12 months | | | |
| Difference (±LSD) | | | |
| LDL-C (mmol/L) (P=x.xxx)[1] | | | |
| Baseline | | | |
| 12 months | | | |
| Difference (±LSD) | | | |
| HDL-C (mmol/L) (P=x.xxx)[1] | | | |
| Baseline | | | |
| 12 months | | | |
| Difference (±LSD) | | | |
| PHQ-9 (P=x.xxx) | | | |
| Baseline | | | |
| 12 months | | | |
| Difference (+LSD) | | | |

[1.] P-value for the F-test of the Time by Treatment interaction.

DRAFT

**Table 3.** Proportions of participants meeting the goals of the Life! Program at baseline and at 12 months, and, total number of goals achieved at 12 months.

| | Control | Intervention | P-value (Treatment main effect and interaction) |
|---|---|---|---|
| No more than 30% energy from fat | | | |
| Baseline | x.xxx (n) | x.xxx (n) | x.xxx |
| 12 months | x.xxx (n) | x.xxx (n) | |
| P-value (Time main effect and interaction) | x.xxx | | x.xxx |
| No more than 10% energy from saturated fat | | | |
| Baseline | x.xxx (n) | x.xxx (n) | x.xxx |
| 12 months | x.xxx (n) | x.xxx (n) | |
| P-value (Time main effect and interaction) | x.xxx | | x.xxx |
| At least 15 g/1,000 kcal fibre intake | | | |
| Baseline | x.xxx (n) | x.xxx (n) | x.xxx |
| 12 months | x.xxx (n) | x.xxx (n) | |
| P-value (Time main effect and interaction) | x.xxx | | x.xxx |
| At least 30 minutes/day moderate intensity | | | |

| | | | |
|---|---|---|---|
| physical activity | | | |
| Baseline | x.xxx (n) | x.xxx (n) | x.xxx |
| 12 months | x.xxx (n) | x.xxx (n) | |
| P-value (Time main effect and interaction) | x.xxx | | x.xxx |
| At least 5% reduction in body weight | | | |
| 12 months | x.xxx (n) | x.xxx (n) | x.xxx |
| Total number of goals achieved | | | |
| 12 months | x.x (n) | x.x (n) | x.xxx |

**Table 4.** Predicted means of primary and secondary endpoints (± 95%CI) for participants in the intervention arm at baseline, 3 months (T2) and 12 months (T3) – males and females

| | Baseline | 3 months | 12 months |
|---|---|---|---|
| Weight (kg) (P= x.xxx) | xx.x (xx.x, xx.x) | xx.x (xx.x, xx.x) | xx.x (xx.x, xx.x) |
| Waist circumference (cm) (P= x.xxx) | | | |
| FPG (mmol/L) (P= x.xxx) | | | |
| Systolic BP (mmHg) (P= x.xxx) | | | |
| Diastolic BP (mmHg) (P= x.xxx) | | | |
| Total cholesterol (mmol/L) (P= x.xxx) | | | |
| Triglycerides (mmol/L) (P= x.xxx) | | | |
| LDL-C (mmol/L) (P= x.xxx) | | | |
| HDL-C (mmol/L) (P= x.xxx) | | | |
| PHQ-9 (P=x.xxxx) | | | |

*Superscripts to be used to denote statistically significance differences between time periods

DRAFT

## Literature Cited

ST Shih, Nathalie Davis-Lameloise, Edward D Janus, Carol Wildey, Vincent L Versace, Virginia Hagger, Dino Asproloupos, Sharleen O'Reilly, Paddy A Phillips, Michael Ackland, Timothy Skinner, Jeremy Oats, Rob Carter, James D Best, James A Dunbar (2013) Mothers After Gestational Diabetes in Australia Diabetes Prevention Program (MAGDA-DPP) post-natal intervention: study protocol for a randomized controlled trial. *Trials*, doi:10.1186/1745-6215-14-339

Nathalie Davis-Lameloise, Andrea Hernan, Edward D Janus, Elizabeth Stewart, Rob Carter, Catherine M Bennett, Sharleen O'Reilly, Benjamin Philpot, Erkki Vartiainen, James A Dunbar and and on behalf of the Melbourne Diabetes Prevention Study (MDPS) research group (2012) The Melbourne Diabetes Prevention Study (MDPS): study protocol for a randomized controlled trial. *Trials*, doi:10.1186/1745-6215-14-31.

DRAFT

## APPENDIX – Derived variables

1. **BMI:**

$$\frac{\text{(Weight in kg)}}{\text{(Height in metres)}^2}$$

2. **Framingham**

$\mu$ = 18.8144 – 1.2146 x female – 1.8443 x log(age) + 0.3668 x log(age) x female – 1.4032 x log(SBP) – 0.3899 x cigarettes – 0.5390 x log(total-C / HDL-C) – 0.3036 x diabetes – 0.1697 x diabetes x female – 0.3362

Where:
Age–age in years
Sex–1 if female, 0 if male
SBP–average of two office measurements of SBP in mm Hg
Total cholesterol–total serum cholesterol in mg/dl
HDL–HDL in mg/dl
Cigarettes–1 if cigarette smoker (or quit within last year), 0 otherwise
Diabetes–1 if diabetes, 0 otherwise
ECG-LVH–1 if definite ECG-LVH, 0 otherwise.

http://www.heartfoundation.org.au/SiteCollectionDocuments/Absolute-risk-technical-report.pdf

3. **The five Life! Program goals and methods used to derive variables from the Food Frequency Questionnaire (DQES v2).**

| Goal | Assumptions and equations used to derive variable for analysis |
|---|---|
| 1. No more than 30% energy from fat; | 1 gram of fat = 9 kcals<br><br>Convert 'All Fat g/day' to kcals (Using the example in Table 6.8 in DQES v2)<br><br>61.17 All Fat g/day *9 = 550.53 kcals<br><br>Convert 'Energy kJ/day' to kcals<br><br>6699.82*0.239 = 1601.26 kcals<br><br>**Conclusion**<br><br>(550.53/1601.26)*100 = 34.38% energy from fat. This participant did not meet the goal of <30% energy from fat. |

DRAFT

| | |
|---|---|
| 2. No more than 10% energy from saturated fat | 1 gram of fat = 9 kcals<br><br>Convert ‘SatFat g/day’ to kcals (Using the example in Table 6.8 in DQES v2)<br><br>30.63 SatFat g/day *9= 275.67<br><br>Convert ‘Energy kJ/day’ to kcals<br><br>6699.82*0.239 = 1601.26<br><br>**Conclusion**<br><br>(275.67/1601.26)*100 = 17.22% energy from saturated fat. This participant did not meet the goal of <10% energy from saturated fat. |
| 3. At least 15 g/1,000 kcal fibre intake | Participant’s Fibre g/day = 19.42<br><br>Participant’s Energy kJ/day = 6699.82<br><br>Convert kJ to kcals<br><br>6699.82*0.239 = 1601.26<br><br>Convert to per/1000<br><br>1601.26/1000 = 1.60<br><br>Divide Fibre intake by 1.60 to get actual fibre intake per 1,000 kcals<br><br>**Conclusion**<br><br>19.42/1.60 = 12.13 g/1,000 kcal. This participant did not meet the goal of at least 15g/1,000 kcal fibre intake.<br><br>This goal can also be derived by multiplying the actual fibre intake in g/day by 1.60 to see how much fibre the participant would have needed to consume to meet the goal:<br><br>19.42 g/day * 1.6 = 24.02 g day |
| 4. At least 30 minutes/day moderate intensity physical activity | Those participants selecting the option ‘Daily’ in the *Physical Activity: Part 5* section will be deemed to have achieved this goal. |
| 5. At least 5% reduction in body weight | $(Weight_{12\ months} – Weight_{Baseline}) / Weight_{Baseline}) * 100$<br><br>Participants with values ≤-5.00% will be deemed to have achieved this goal. |

DRAFT

DRAFT
