# Supplementary figures and images for: Mothers after Gestational Diabetes in Australia (MAGDA): A Randomised Controlled Trial of a Postnatal Diabetes Prevention Program
Source: PLoS Med. 2016 Jul 26;13(7):e1002092. doi: 10.1371/journal.pmed.1002092 (PMC4961439; doi:10.1371/journal.pmed.1002092)

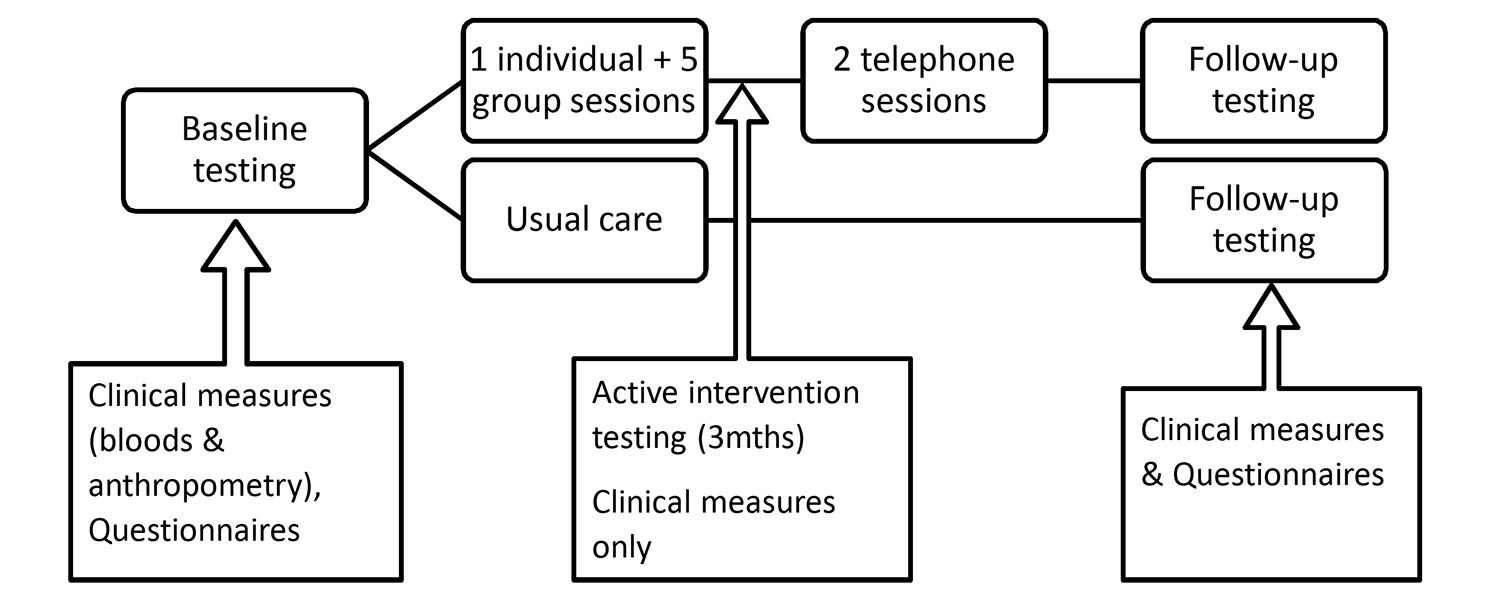

Supplement: S1 Fig — (TIF) [file pmed.1002092.s005.tif]

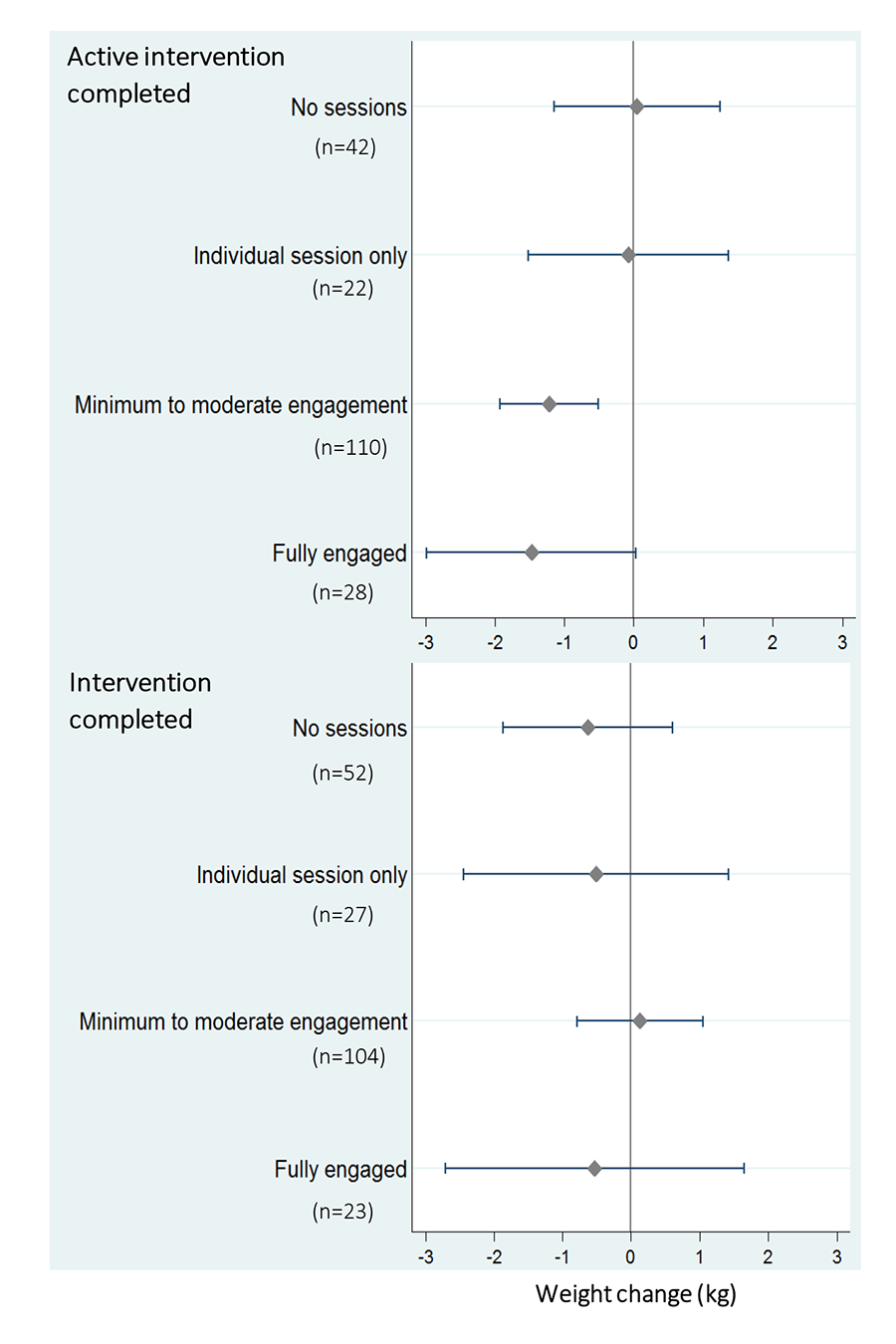

Supplement: S2 Fig — Minimum to moderate engagement was defined as attending the individual session and 1–4 group sessions; full engagement was attending all sessions. (TIF) [file pmed.1002092.s006.tif]
